# Quantitative Analysis of a Novel Metabolite Panel to Estimate GFR (Panel eGFR) in Serum and Plasma Using LC-MS/MS

**DOI:** 10.1093/clinchem/hvaf110

**Published:** 2025-10-10

**Authors:** Nora Fino, Lesley A Inker, Seiei Shiba, Ogechi M Adingwupu, Josef Coresh, Ben Haaland, Michael G Shlipak, Andrew Levey, Jesse C Seegmiller, The Multi-Ethnic Study of Atherosclerosis (MESA-Kidney) Study Group, Tariq Shafi, Wendy Post, Peter Rossing, The HIV Study Group, Christina Wyatt, Zipporah Krishnasami, James Hellinger

**Affiliations:** Division of Biostatistics, Department of Population Health Sciences, University of Utah Health, Salt Lake City, UT, United States; Department of Medicine, Division of Nephrology, Tufts Medical Center, Boston, MA, United States; Department of Laboratory Medicine and Pathology, University of Minnesota, Minneapolis, MN, United States; Department of Medicine, Division of Nephrology, Tufts Medical Center, Boston, MA, United States; Department of Population Health, NYU Langone, New York, NY, United States; Division of Biostatistics, Department of Population Health Sciences, University of Utah Health, Salt Lake City, UT, United States; Kidney Health Research Collaborative, San Francisco Veterans Affair Medical Center and University of California, San Francisco, CA, United States; Department of Medicine, Division of Nephrology, Tufts Medical Center, Boston, MA, United States; Department of Laboratory Medicine and Pathology, University of Minnesota, Minneapolis, MN, United States

## Abstract

**Background:**

Estimated glomerular filtration rate (eGFR) using creatinine (eGFRcr), cystatin C (eGFRcys), or both (eGFRcr-cys) is not sufficiently accurate in many settings, often due to non-glomerular filtration rate (GFR) determinants of the filtration markers. In principle, using a panel of endogenous markers (panel eGFR) could reduce the impact of non-GFR determinants of each marker, improving the accuracy of eGFR. Using global untargeted metabolomics, we previously identified 33 endogenous metabolites that correlate highly with measured GFR.

**Methods:**

A LC-MS/MS measurement procedure was developed to quantify 11 endogenous metabolites from serum and plasma. The assay was evaluated in 99 participants with measured GFR (mGFR) from 2 research studies, including a subgroup of 51 participants with large errors in eGFRcr and large discordance between eGFRcr and eGFRcys. Performance of eGFR models using single metabolites and all metabolites (panel eGFR-11) compared to mGFR was assessed by leave-one-out cross-validated root mean square error (RMSE).

**Results:**

Assay CV for single metabolites ranged from 1.1% to 6.3% over the course of 21 days. RMSE of eGFR in single metabolite models ranged from 0.184 to 0.324. RMSEs for panel eGFR-11, eGFRcr, and eGFRcr-cys were 0.195, 0.251, and 0.201, respectively, and 0.155, 0.290, and 0.203, respectively, in the subgroup with large errors and large discordance.

**Conclusions:**

A precise metabolite (LC-MS/MS) measurement procedure shows promise for more accurate GFR estimation when eGFRcr is unreliable, offering a potential new confirmatory test for GFR evaluation.

## Introduction

Glomerular filtration rate (GFR) is widely regarded as the best overall index of kidney function in health and disease (1, 2). Although GFR can be measured using the clearance of exogenous filtration markers [measured GFR (mGFR)], these procedures are complex and not widely available. As a result, standard clinical practice for GFR evaluation relies on estimated GFR (eGFR) computed from serum creatinine concentration (eGFRcr), together with age and sex. When eGFRcr is suspected to be inaccurate or a more accurate evaluation is needed, guidelines recommend using eGFR from serum cystatin C concentration (eGFRcys) or a combination of creatinine and cystatin C concentrations (eGFRcr-cys)(3, 4). However, for both eGFRcr and eGFRcys, large errors of greater than 30% relative to mGFR have been observed in up to 10%–20% of subjects in populations included in validation studies and in up to 40% in patients with chronic conditions in addition to kidney disease, where clinical decisions based on GFR have the highest impact (5, 6).

Inaccuracy in eGFR is largely attributed to non-GFR determinants of the serum concentrations of endogenous filtration markers (such as muscle mass, diet, or medications for creatinine and obesity, smoking, and inflammation for cystatin C) that differ from the populations used to develop the eGFR equations (1, 7). We have proposed that using a panel of multiple endogenous filtration markers with different non-GFR determinants (panel eGFR) could minimize the impact of non-GFR determinants of each single marker (1, 8, 9), thereby improving accuracy and potentially eliminating the need to include demographic factors.

In previous work, we developed prototype versions of panel eGFR (8, 10–12). These prototypes did not consistently improve accuracy in comparison to eGFRcr-cys, in part because of a lack of a sufficient number of candidate filtration markers. From global untargeted metabolomic studies, we identified 33 candidate metabolite filtration markers with high negative correlations to mGFR, many of which were not previously known as filtration markers (9). In this report, we first describe the development of a targeted LC-MS/MS measurement procedure using a subset of metabolites that met analytical validation criteria. We then describe the initial clinical performance of models for eGFR using this LC-MS/MS procedure in 2 clinical populations.

## Materials and Methods

The supplement contains additional information on study design and analytical and procedural details.

### Study Design

We developed and validated an LC-MS/MS measurement procedure for metabolites that exhibited clinically acceptable analytical performance. We used stored samples from 2 cohorts for preliminary clinical validation. The human immunodeficiency virus (HIV) study evaluated the performance of eGFR compared to mGFR in 200 adults with HIV on stable antiretroviral therapy (13). Serum samples from 43 participants were included. The Multi-Ethnic Study of Atherosclerosis (MESA) is a community-based, multiethnic prospective study of adults with subclinical cardiovascular disease. MESA-Kidney performed mGFR in a subgroup of 294 participants. Serum, EDTA plasma, and heparin plasma samples from 56 participants were included (14). The institutional review boards for the HIV study and MESA-Kidney approved these studies, and the institutional review board at Tufts Medical Center approved the overall analyses.

### Development and Analytical Validation of the LC-MS/MS Measurement Procedure

#### Reagents and Instrumentation, Calibrator, Internal Standard, and Quality Control Preparation

A complete description of the development of the calibrator and quality control (QC) samples including the concentrations along with chemical and material sources are provided in the supplement (Reagent section and Supplemental Tables 1–4). In brief, 1 mg/mL stock solutions were created for all compounds in 50% acetonitrile, aside the kynurenic acid stock, which was made with 0.1% ammonium hydroxide in 50% acetonitrile. The intermediate calibrator stock solution was prepared by adding stock solutions into 5% w/v bovine serum albumin in phosphate-buffered saline. Nine calibrators were prepared by spiking the intermediate calibrator stock solution into 5% w/v bovine serum albumin in phosphate-buffered saline. The working internal standard solution was created from 1 mg/mL of each isotopically labeled compound into 50% acetonitrile (Supplemental Table 4). Low-, medium-, and high-QCs were prepared by adding 0.5 to 10 mL of the intermediate calibrator stock solution into stripped serum. All calibrators and controls were aliquoted as 0.40 mL and frozen at −80°C until used.

#### Sample Preparation

The working calibrators, QCs, and specimens were thawed at 25°C for 1 hour and mixed by inversion 10 times prior to preparation. A 100 µL from all calibrators, controls, and specimens were processed by adding 20 µL of internal standard solution followed by 400 µL acetonitrile with 0.1% formic acid. A 200 µL aliquot of the supernatant was dried down using a Vacufuge plus Centrifuge Concentrator at 45°C for 2 hours and reconstituted in 200 µL of 5% acetonitrile with 0.1% formic acid and then transferred to autosampler vials (Chrome Tech) for analysis.

#### LC-MS/MS Conditions

Analysis was performed by LC-MS/MS using a Shimadzu XR LC system (Shimadzu Corporation) coupled with a SCIEX QTRAP 6500 triple quadrupole detector (AB Sciex). For each run, 10 µL of processed sample was injected onto a 100 × 4.6 mm Synergi™ 4 µm Hydro-RP 80 Å LC Column from Phenomenex. Chromatography was carried out at 800 µL/min over 8 min. The mobile phases comprised of solvent A (0.1% formic acid in water) and solvent B (0.1% formic acid in acetonitrile). The chromatographic run began at 2% B and was held for 2 min, followed by a 3-min linear gradient to 60% B stepped to 95% B over 0.1 min, held for 0.8 min, stepped to 2% B in 0.1 min, and held for 2 min. Transitions were monitored using polarity switching and scheduled multiple reaction monitoring with a 40-s scheduled multiple reaction monitoring detection window and a 0.20-s target cycle time.

Ion suppression was evaluated by the post-column infusion technique whereby extracted samples were injected while continuously infusing compounds at 100 ng/mL dissolved in 50% methanol at 10 µL/min post-column. Interference studies were conducted using the Assurance Interference Test Kit (Sun Diagnostics) by spiking 1200 mg/dL triglyceride-rich lipoproteins, 800 mg/dL hemolysate, and 32 mg/dL conjugated bilirubin into a heparin plasma pool.

Data were acquired using SCIEX Analyst (Version 1.7.1) and processed using SCIEX MultiQuant software (Version 3.0.2) with the SignalFinder algorithm for kynurenic acid and MQ4 algorithm for all the other metabolites. The analyte concentrations were determined by comparing the ratio of the peak area to the corresponding internal standard or surrogate internal standard peak area and referencing to an external calibration curve using inverse-weighted linear regression analysis. Area ratios were assessed for all compounds with a limit of ±20%, aside from symmetric dimethylarginine and N-acetyl-L-alanine, where only one multiple reaction monitoring transition per metabolite was observed (Supplemental Table 5). Accuracy was assessed for every run at ≤±15% of the expected value for calibrators and control and ≤±20% at the limit of quantitation.

Metabolite linearity was assessed through 8 serial dilutions from the highest calibrator concentration added to a stripped serum matrix for all analytes. Interassay and intraassay imprecision were determined by analyzing the 3 QC samples for 21 days and 20 times within a single run, respectively. Recovery was assessed by spiking specimens from all collection tube types with intermediate calibrator stock solution at low, medium, and high concentrations. Carryover was assessed by comparing the average of 3 injections of a low calibrator followed by 3 injections of the highest calibrator and one injection of the low calibrator and referencing this result to the average of the low calibrator.

Specimen type comparison studies were conducted across samples collected in serum, EDTA plasma, and lithium heparin plasma collection tubes from 5 healthy donors and from 56 MESA-Kidney study participants. For the MESA-Kidney specimens, agreement was assessed by CV and Deming regression, before and after removing outliers, defined as more than ±3 SDs above or below the metabolite's mean value.

Using donor specimens, stability was assessed for all collection tube types by comparing results from the collection day. Comparison was made for aliquots stored at 23°C, 4°C, −20°C, and −80°C on day 0, 1, 3, 7, and 28. The aliquots stored at −20°C, and −80°C had an additional aliquot to perform a 3-cycle freeze-thaw study.

### Clinical Validation

#### Overall Approach

Our goal was to develop a metabolite panel eGFR providing more accurate GFR estimates in settings where current clinical estimates are insufficiently accurate for clinical decisions. Thus, in the design for this initial validation of panel eGFR, we focused on individuals with large errors in eGFRcr compared to mGFR and large discordances between eGFRcr and eGFRcys. In principle, this design would be advantageous for comparisons of the panel to eGFRcys and disadvantageous for comparisons of the panel to eGFRcr.

Chronic Kidney Disease Epidemiology Collaboration equations were used to estimate GFR (6). We defined errors in eGFR as the absolute percent difference between mGFR and eGFR, relative to mGFR. Errors were defined as greater than or equal to 30% and the percentage of individuals with large errors (1-P_30_). Discordance between eGFRcr and eGFRcys was defined as the absolute difference between the 2 estimates (eGFRdiff) exceeding 15 mL/min/1.73 m^2^. For the clinical validation dataset, participants with discordance between eGFRcr and eGFRcys were included to ensure precise estimates for this key group. The remaining patients were randomly sampled for a total of 99 participants, a sample size determined by feasibility of performing the measurements (Supplemental Fig. 1).

#### Creatinine, Cystatin C, and mGFR Determinations

The supplement contains information on creatinine and cystatin C assays. GFR was measured using plasma clearance of iohexol and expressed with indexing to body surface area in both the HIV and MESA-Kidney studies, as previously described (13, 14).

#### Statistical Analysis

In all analyses, we accounted for the sampling design by weighting each observation by the inverse probability of being sampled. Pearson correlations between the markers and mGFR, serum creatinine, and serum cystatin C were summarized overall and by sampling group on the log scale. For MESA-Kidney, we compared the Pearson correlations between the values for the individual metabolites obtained using the LC-MS/MS procedure and mGFR to our prior results for the correlations between the relative values for each metabolite obtained from the global untargeted metabolomics and mGFR (14).

Ordinary least squares linear regression was used to develop models for estimating GFR for each metabolite (panel eGFR-1) and the combination of all 11 metabolites (panel eGFR-11). Effects of adding age, sex, body mass index (BMI), and race group (Black vs non-Black) were examined. All panel eGFR models were compared to eGFRcr, eGFRcys, and eGFRcr-cys equations tailored to this dataset by refitting the variables in the Chronic Kidney Disease Epidemiology Collaboration equations while keeping the knot locations for the serum creatinine and serum cystatin C spline terms unchanged.

The model performance was evaluated using 2 metrics: root mean square error (RMSE) and the percentage of individuals with large errors (1-P_30_). These metrics were calculated using leave-one-out cross-validation (15). Performance metrics were calculated in the overall group and within each sampling group.

## Results

### Analytical Validation

**Fig. 1.**
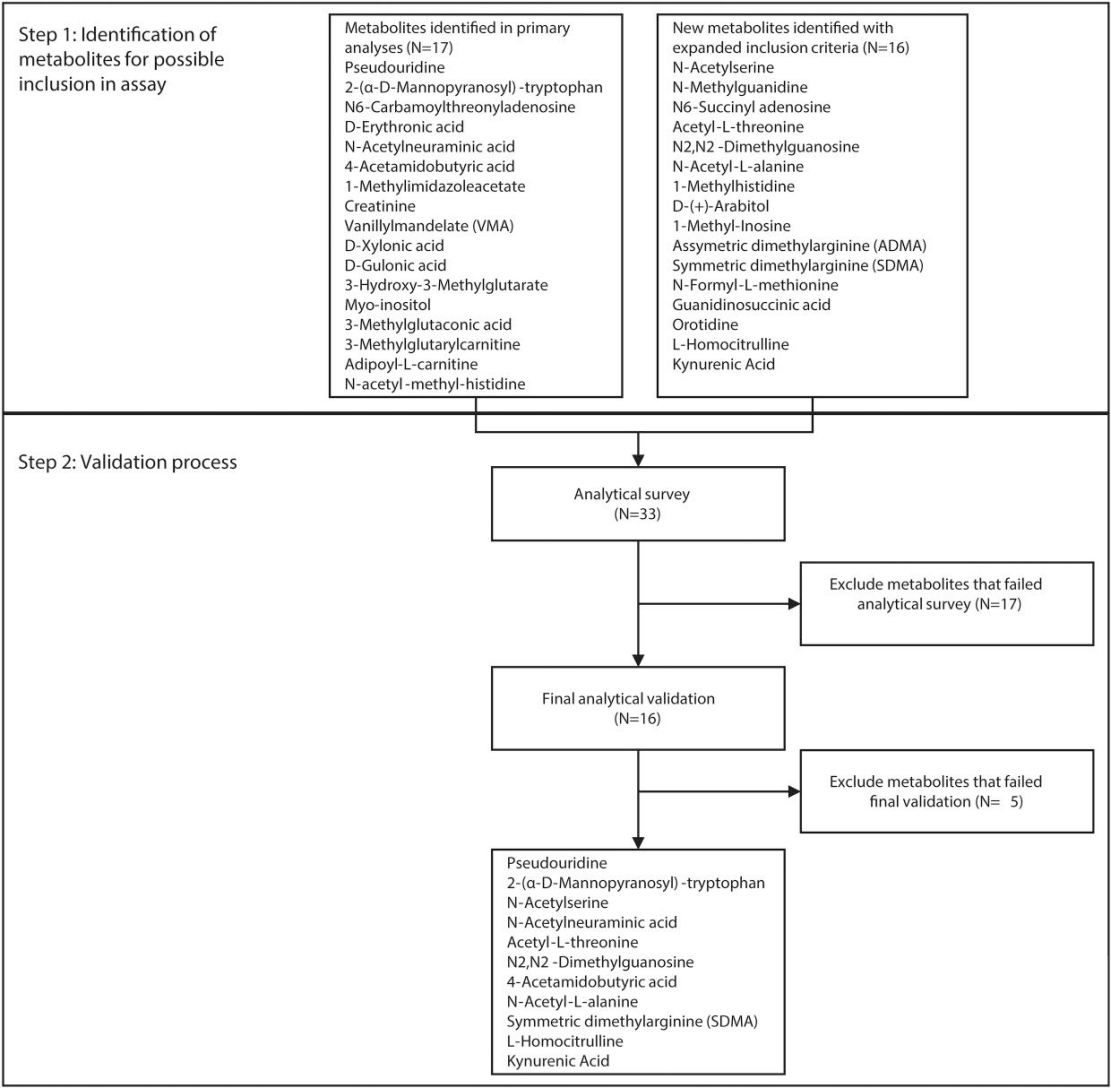
Summary of metabolites during identification and validation phases. Using results from the global metabolomics approach, we performed preliminary analysis for metabolite selection, which produced 16 top metabolites listed in the left box (9). The analyses were repeated with an expanded criteria, and new metabolites (right box) were identified resulting in a list of 33 metabolites for analytical assessment.

Among the 33 metabolites considered, 16 passed analytical survey tests (Fig. 1). Of the remaining 16 compounds, 5 failed analytical validation due to various issues, including ion suppression/enhancement, chromatographic interference, poor recovery, or inadequate linearity.

#### Limit of Quantitation and Limit of Detection

Each metabolite met the requirement of a CV of ≤20% and accuracy of ≤ ±20% (Supplemental Table 6) when assessing the calibrators over 21 separate days. Supplemental Fig. 2 shows representative calibrator and specimen chromatograms.

#### Ion Suppression/Interference Studies

Ion enhancement was observed for symmetric dimethylarginine, kynurenic acid, and N-acetylneuraminic acid at 30%, 15%, and 25%, respectively. Ion suppression was observed for L-homocitrulline by 25%. However, we found the observed recoveries were satisfactory, falling between 85% and 115%.

#### Imprecision, Linearity, Recovery, and Carryover

The interassay CVs across the 3 QCs were ≤6.3% (Table 1). Linear calibration was observed over a 200-fold range with the coefficient of determination (*R*^2^ > 0.99). Metabolite linearity was assessed through 8 serial dilutions from the highest calibrator concentration added to a stripped serum matrix (Supplemental Table 7). Linear regression slopes were between 0.9890 and 1.01 with *R*^2^ > 0.999 for all analytes, and the observed calibration results were ≤±15% and <±20% difference at the limit of quantitation. Mean recoveries were observed from 95.5% to 111% in all sample matrices (Supplemental Table 8). The carryover was <11% when assessed after the highest calibrator for all analytes.

**Table 1.** Measurement Procedure LLOQ and ULOQ and interassay imprecision for 3 QCs.

	LLOQ (µM)	ULOQ (µM)	QC concentration (µM)			Interassay CV N = 21 (%)		
			Low	Med	High	Low	Med	High
Pseudouridine	0.25	50	1.208	5.034	24.791	1.7	1.7	1.6
2-(**α**-D-Mannopyranosyl)-tryptophan	0.02	4	0.100	0.394	1.979	6.2	5.7	4.6
Symmetric dimethylarginine	0.05	10	0.245	1.017	5.059	3.9	3.1	2.5
L-Homocitrulline	0.05	10	0.236	0.985	4.885	3.3	2.1	2.3
Kynurenic acid	0.006	1.2	0.029	0.120	0.594	4.2	2.1	1.7
N^2^,N^2^-Dimethylguanosine	0.006	1.2	0.030	0.119	0.592	5.3	3.2	1.9
N-Acetylserine	0.15	30	0.748	3.046	14.846	3.0	2.6	2.1
N-Acetylneuraminic acid	0.1	20	0.470	1.977	9.699	6.3	3.1	3.3
Acetyl-L-threonine	0.06	12	0.293	1.200	5.912	2.1	1.3	1.6
N-Acetyl-L-alanine	0.1	20	0.501	2.049	9.993	2.0	1.3	1.1
4-Acetamidobutanoic acid	0.05	10	0.253	1.033	5.091	1.8	1.9	1.7

Abbreviations: LLOQ, lower limit of quantification; ULOQ, upper limit of quantification.

#### Stability

Result bias from the day 0 collection result varied by −5.2% to 12.3% at 23°C, −5.8% to 11.3% at 4°C, −8.3% to 7.4% at −20°C, and −5.0% to 7.5% at −80°C over 28 days for all analytes. Result bias from 3 freeze-thaw cycles ranged from −7.0% to 10.5% at −20°C and from −8.5% to 9.8% at −80°C for all analytes. Additional stability studies on the QCs material where the bias ranged from −3.2% to 9.2% at 4°C, from −4.0% to 8.7% at −20°C, and from −4.8% to 8.6% at −80°C over 28 days when compared to the day 0 results. Evaluation of 3 freeze-thaw cycles of the QC varied from −2.7% to 5.3% at −20°C and −4.8% to 6.7% at −80°C when compared to the day 0 QC results.

#### Agreement across Collection Tubes

In the 5 donors and the MESA-Kidney participants, we observed agreement in the values for all of the individual metabolites across the serum, EDTA plasma, and lithium heparin plasma collection tubes (Supplemental Tables 8–10).

### Clinical Validation

#### Study Population

The characteristics of the participants selected for clinical validation and the subgroup with large errors in eGFRcr and large eGFRdiff are presented in Table 2. A comparison with the remainder of the individuals in the parent study populations is provided in Supplemental Table 11. In the subgroup with large errors in eGFRcr and large eGFRdiff, mGFR was generally lower than the overall population, with mean 67.8 (SD 24.4) mL/min/1.73 m^2^.

**Table 2.** Descriptive characteristics of the clinical validation datasets, overall and in the subset of individuals with large errors in eGFRcr and large discordance between eGFRcr and eGFRcys (large eGFRdiff).^a^

	Overall^b^	Large error in eGFRcr and large eGFRdiff^c^
*n*	99	51
MESA-Kidney participant (%)	(55.8)	32 (62.7)
Age, years	61.1 (16.3)	65.4 (16.0)
Black race (%)	(49.5)	17 (33.3)
Female sex (%)	(45.3)	24 (47.1)
Diabetes (%)	(11.9)	11 (21.6)
BMI, kg/m^2^	28.1 (5.0)	29.0 (7.4)
Serum creatinine, mg/dL	1.0 (0.3)	1.1 (0.4)
Serum cystatin-c, mg/L	1.0 (0.2)	1.2 (0.3)
mGFR, mL/min/1.73 m^2^	80.3 (25.1)	67.8 (24.4)
eGFRcr, mL/min/1.73 m^2^	78.3 (21.7)	73.8 (15.1)
eGFRcys, mL/min/1.73 m^2^	79.2 (24.4)	64.4 (23.8)
eGFRcr-cys, mL/min/1.73 m^2^	81.7 (22.4)	69.5 (15.5)
Pseudouridine, µM	3.79 (0.97)	4.61 (1.22)
2-(alpha-D-mannopyranosyl)-tryptophan, µM	0.21 (0.06)	0.27 (0.08)
Symmetric dimethylarginine, µM	0.51 (0.11)	0.61 (0.13)
L-homocitrulline, µM	0.44 (0.40)	0.51 (0.32)
Kynurenic acid, µM	0.04 (0.02)	0.06 (0.04)
N2,N2-dimethylguanosine, µM	0.04 (0.01)	0.05 (0.02)
N-acetylserine, µM	1.18 (0.42)	1.46 (0.93)
N-acetylneuraminic acid, µM	0.76 (0.21)	0.88 (0.32)
Acetyl-L-threonine, µM	0.69 (0.20)	0.85 (0.29)
N-acetyl-L-alanine, µM	1.48 (0.34)	1.72 (0.59)
4-acetamidobutanoic acid, µM	0.19 (0.09)	0.26 (0.15)

^a^Data are presented as n (%) or mean (SD).

^b^Overall statistics account for the sampling design.

^c^Large error in eGFRcr is defined as an absolute percent difference between mGFR and eGFRcr relative to mGFR that exceeds 30%. Large eGFRdiff is defined as an absolute difference in eGFRcr and eGFRcys greater than 15 mL/min/1.73 m^2^.

#### Correlation of Metabolites with mGFR

Correlations of all metabolites and cystatin C with mGFR were below −0.5, except for creatinine (−0.36) and kynurenic acid (−0.38) (Table 3). The strongest correlations were observed with 2−(alpha-D-mannopyranosyl)-tryptophan (*r* = −0.81), symmetric dimethylarginine (*r* = −0.71), pseudouridine (*r* = −0.70), and 4-acetamidobutanoic acid (*r* = −0.71). Notably, in the subgroup, each of these metabolites maintained high correlations with mGFR, ranging from −0.92 to −0.67, whereas creatinine displayed a positive correlation.

**Table 3.** Correlations between the markers and mGFR on the log scale, overall, and in the subset of individuals with large error and large eGFRdiff.^a^

Log transformed biomarker	Overall^b^ (n = 99)	Large error in eGFRcr and large eGFRdiff^c^ (n = 51)
Serum creatinine	−0.36	0.33
Serum cystatin-c	−0.66	−0.87
Pseudouridine	−0.70	−0.87
2-(alpha-D-mannopyranosyl)-tryptophan	−0.81	−0.92
Symmetric dimethylarginine	−0.71	−0.75
L-homocitrulline	−0.55	−0.39
Kynurenic acid	−0.38	−0.25
N2,N2-dimethylguanosine	−0.65	−0.64
N-acetylserine	−0.58	−0.58
N-acetylneuraminic acid	−0.67	−0.72
Acetyl-L-threonine	−0.64	−0.65
N-acetyl-L-alanine	−0.64	−0.60
4-acetamidobutanoic acid	−0.70	−0.67

^a^Correlations were calculated after log-transformation of markers and mGFR. Large error refers to large errors in eGFRcr and large eGFRdiff refers to large differences in eGFRcr and eGFRcys.

^b^Overall correlations account for the sampling design.

^c^Large error in eGFRcr is defined as an absolute percent difference between mGFR and eGFRcr, relative to mGFR, that exceeds 30%. Large eGFRdiff is defined as an absolute difference in eGFRcr and eGFRcys greater than 15 mL/min/1.73 m^2^.

When comparing the correlations of metabolites with mGFR between the global metabolomics and the LC-MS/MS procedure in MESA-Kidney (Supplemental Table 12), no consistent pattern was observed. Some associations with mGFR were notably stronger with results from the LC-MS/MS procedure, such as pseudouridine (*r* = −0.76 vs *r* = −0.60) and symmetric dimethylarginine (*r* = −0.71 vs *r* = −0.50). Others were similar or weaker, such as 2-(alpha-D-mannopyranosyl)-tryptophan (*r* = −0.80 in both) or N-acetylneuraminic acid (*r* = −0.37 vs *r* = −0.62).

#### Performance

Table 4 presents the leave-one-out cross-validated performance of models using eGFRcr, eGFRcys, eGFRcr-cys, and individual metabolites. In the overall cohort, eGFRcr, eGFRcys, and eGFRcr-cys had a RMSE of 0.251, 0.194, and 0.201, respectively, and 1-P_30_ of 26.3%, 12.1%, and 14.1%, respectively. In single-metabolite models for eGFR, RMSEs and 1-P_30_ ranged from 0.184 and 10.1% for 2-(alpha-D-mannopyranosyl)-tryptophan to 0.324 and 42.4% for kynurenic acid. For panel eGFR-11, RMSE and 1-P_30_ were 0.195 and 13.3%.

**Table 4.** Performance of GFR estimating equations using novel metabolites, overall and in the subset of individuals with large error and large eGFRdiff.^a^

	Overall (n = 99)		Large error in eGFRcr and large eGFRdiff^e^ (n = 51)	
	RMSE	1-P_30_ (%)	RMSE	1-P_30_ (%)
Reference equation^b^				
eGFRcr	0.251	26.3	0.290	41.20
eGFRcys	0.194	12.10	0.153	7.80
eGFRcr-cys	0.201	14.10	0.203	13.70
Models using novel metabolites				
Panel eGFR-1^c^				
Pseudouridine	0.220	15.20	0.175	3.90
2-(alpha-D-mannopyranosyl)-tryptophan	0.184	10.10	0.133	2.00
Symmetric dimethylarginine	0.243	17.20	0.225	15.70
L-homocitrulline	0.303	34.30	0.328	43.10
Kynurenic acid	0.324	42.40	0.335	51.00
N2,N2-dimethylguanosine	0.258	27.30	0.255	25.50
N-acetylserine	0.284	35.40	0.285	37.30
N-acetylneuraminic acid	0.259	28.30	0.241	31.40
Acetyl-L-threonine	0.259	28.30	0.252	33.30
N-acetyl-L-alanine	0.277	27.30	0.275	29.40
4-acetamidobutanoic acid	0.237	22.20	0.247	25.50
Panel eGFR-11^d^	0.195	13.30	0.155	5.90

^a^All models were developed in the overall sample and accounted for the sampling design. Performance metrics were calculated using leave-one-out cross-validation. Models using the novel metabolites include only the listed metabolites (i.e., they do not contain demographic variables age, sex, BMI, or race).

^b^The reference equation eGFRcr-cys used the form of the Chronic Kidney Disease Epidemiology equations using creatinine and cystatin C and was refit to the study population.

^c^Panel eGFR-1 models are based on linear models with a single given metabolite.

^d^Panel eGFR-11 is based on a linear model with all 11 markers.

^e^Large error in eGFRcr is defined as an absolute percent difference between mGFR and eGFRcr relative to mGFR that exceeds 30%. Large eGFRdiff is defined as an absolute difference in eGFRcr and eGFRcys greater than 15 mL/min/1.73 m^2^.

In the subgroup, 2-(alpha-D-mannopyranosyl)-tryptophan was again the most accurate marker (RMSE 0.133 and 1-P_30_ 2.0%), followed by pseudouridine (RMSE 0.175 and 1-P_30_ 3.9%). Panel eGFR-11 had an RMSE of 0.155 and 1-P_30_ 5.9%, substantially outperforming eGFRcr and eGFRcr-cys (RMSE 0.290 and 0.203, with 1-P_30_ of 41.2% and 13.7%, respectively) and performing similarly to eGFRcys (RMSE 0.153 and 1-P_30_ 7.8%).

**Fig. 2.**
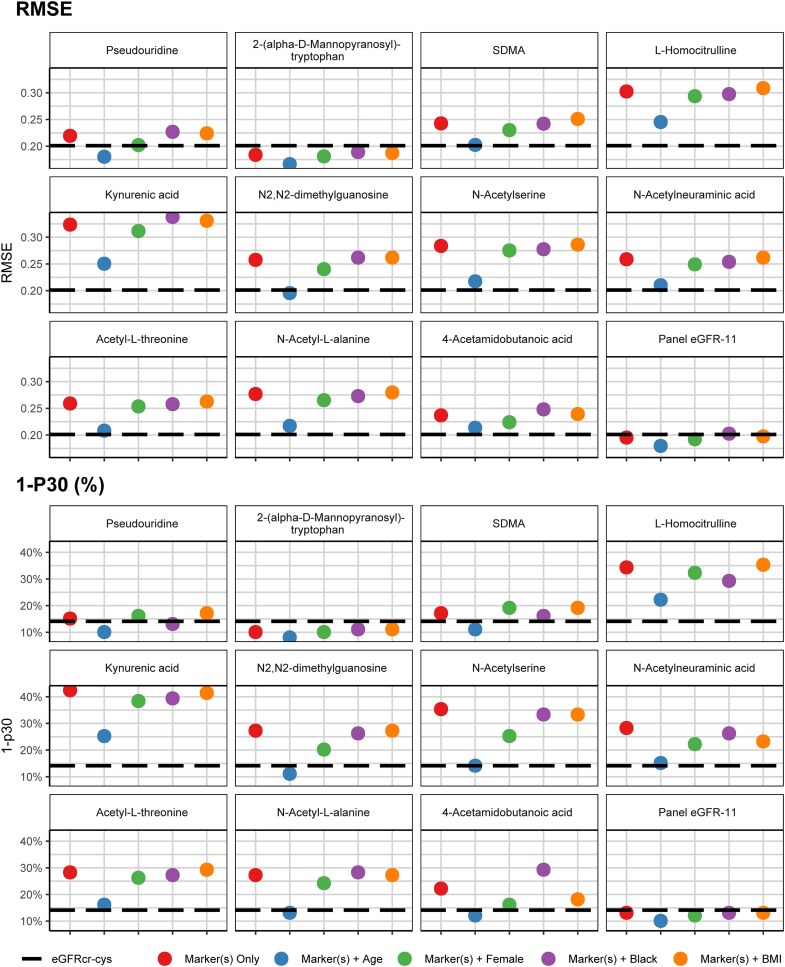
Impact of adding individual demographic terms to single marker panel eGFR-1 models and the multimarker panel eGFR-11 model on RMSE and 1-P_30_ in the overall sample. Performance metrics were calculated using leave-one-out cross-validation. All panel eGFR models were developed in the overall sample and accounted for the sampling design. The dashed line is based on the Chronic Kidney Disease Epidemiology equation using creatinine and cystatin C refit to the study population. The color dots represent a different demographic variable as indicated. Color figure available at clinchem.org.

Figure 2 illustrates the performance of models incorporating metabolites alone or in combination with demographic factors such as age, sex, race, and BMI. In general, the accuracy of single-metabolite models improved slightly when age was included but showed little to no improvement with the addition of sex, race, or BMI. For instance, the RMSE for pseudouridine decreased from 0.220 without demographic adjustments to 0.180 with the inclusion of age. Including sex generally led to a slight improvement in accuracy, while race and BMI typically did not enhance accuracy. For pseudouridine, the RMSE was 0.202 with sex included, compared to 0.227 with Black race and 0.224 with BMI. For panel eGFR-11, adding demographic factors had little to no impact on accuracy (RMSE with no demographics was 0.193 while RMSEs with demographics ranged from 0.180–0.203). Results were largely similar among the subgroup with large errors in eGFRcr and large eGFRdiff (Supplemental Fig. 3).

## Discussion

This paper describes the development and analytical validation of a LC-MS/MS measurement procedure that quantifies 11 endogenous metabolite filtration markers in blood with imprecision of ≤6.3% CV in pooled controls over a 21-day period for all metabolites. Results from linearity and spike recovery studies suggest that the use of a nonhuman matrix calibration system for the analysis of these novel endogenous markers found in humans is a viable approach for this measurement procedure. In an initial exploration of the clinical validation, combining metabolites into a panel eGFR-11 had an RMSE of 0.195 and 1-P_30_ of 13.3%. In the subgroup with large errors in eGFRcr and large discordance between eGFRcr and eGFRcys, in which the performance of eGFRcr is expected to be poor and the performance of eGFRcys is expected to be good, the accuracy of panel eGFR-11 was better than eGFRcr or eGFRcr-cys and similar to eGFRcys. Together, the analytical and clinical validation identify a highly promising pathway to develop a panel eGFR for use as a confirmatory test for GFR evaluation that can be obtained with a single blood sample.

Initially, we evaluated 33 candidate metabolites, but the final measurement procedure included 11 that were most suitable for clinical analysis (9). Key factors influencing inclusion in the final panel were based on analytical performance. Individual metabolites may perform better with alternative processing or chromatographic approaches than those used in this study. Our goal here was to quantitatively multiplex all compounds into a single injection. As a result, while the excluded compounds did not meet the criteria for this specific approach, their utility and correlation with GFR could potentially be enhanced using different analytical approaches.

Errors in eGFR primarily stem from non-GFR determinants of endogenous filtration markers (1). Previous studies have hypothesized that combining multiple filtration markers with largely independent non-GFR determinants into a panel could enhance accuracy, particularly in settings where current filtration markers are inadequate (8–10, 16). This strategy could reduce dependence on any single marker and limit the influence of non-GFR factors on GFR estimation. In current practice, understanding non-GFR determinants of both creatinine and cystatin C is critical to interpretation (3). We anticipate that the use of a panel eGFR would diminish the need to understand the non-GFR determinants of individual markers. Using a separate set of markers, we recently demonstrated that down-weighting of 1 or 2 influential outlier markers decreased the error of the panel eGFR, leading to another method to improve the accuracy of a multimarker panel eGFR (17).

Our previous investigations into multimarker panels did not consistently yield GFR estimates that were significantly more accurate than current methods but had critical differences from the current study (8, 10, 11). Most importantly, previous panels included only 4 markers. In addition, the panel of low molecular weight proteins included creatinine and therefore would not provide an independent confirmation of eGFRcr, the initial test in most settings (10). The novel metabolite panel included tryptophan, which was positively correlated with measured GFR, and thus reflects different pathophysiology than decreased filtration of the marker (11). In contrast, in the present study, all 11 metabolites were inversely correlated with measured GFR. Two metabolites had correlations with mGFR less than 0.5; this might indicate that these are less optimal filtration makers to include in the panel. Future investigations can explore both the biology of these markers as well as the statistical requirements for all 11 to optimize GFR in varied populations (18, 19).

We did not incorporate cystatin C into our panel of filtration markers a priori. Given that creatinine and cystatin C are already widely used, an ideal confirmatory test would exclude both markers (3). Furthermore, given that it is a protein, cystatin C could not be included in the same measurement procedure as these metabolites. Multiple prior studies have shown eGFRcys is not more accurate than eGFRcr and not as accurate as eGFRcr-cys (3), so the finding that eGFRcys was more accurate than eGFRcr-cys and more accurate than panel-eGFR-11 in this study may represent the sampling design of our study and may not be generalizable. Future analyses will assess whether incorporating creatinine or cystatin C within a confirmatory panel eGFR meaningfully improves its accuracy.

Strengths of our study included representation from both Black and White individuals, as well as male and female participants, to increase the generalizability of our findings. Additionally, the HIV study was conducted before the introduction of cobicistat and integrase strand inhibitors, which are known to interfere with tubular secretion of creatinine. However, several limitations warrant consideration. As discussed earlier, we limited our validation to reversed-phase chromatography; other chromatographic techniques might be more appropriate for some of the compounds excluded from our analysis. Stability studies could not be completed in the previously collected clinical samples. Our clinical validation was constrained by a small sample size; while we oversampled a key group of interest, other subgroups remained small. Consequently, these results should be viewed as a preliminary step toward the development of a panel eGFR rather than validation of a specific equation.

In conclusion, we present here a LC-MS/MS measurement procedure for 11 novel metabolites. Future work will evaluate the metabolites using additional samples from diverse patient populations, along with developing novel methods that can both capture complex nonlinear relationships and interactions among the markers as well as yield a customized panel eGFR to individual patients.

## Data and Code Availability

The code for the clinical validation is available at https://github.com/norafino/panel_GFR and at https://figshare.com/articles/journal_contribution/R_Code_for_i_Quantitative_Analysis_of_a_Novel_Metabolite_Panel_to_estimate_GFR_panel_eGFR_in_Serum_and_Plasma_using_Liquid_Chromatography_Tandem_Mass_Spectrometry_LC-MS_MS_i_i_i_Clinical_Chemistry_2025_/29945036?file=57299192.

The Chronic Kidney Disease Epidemiology Collaboration (CKD-EPI) received all data used in this study from third-party sources. As per data use agreements, CKD-EPI does not have the rights to share or legally distribute third-party data. Direct sharing of these data by CKD-EPI would breach data compliance policies approved by our research ethics board. Researchers interested in data used in this study could request access through the following means: Data of Multi-Ethnic Study of Atherosclerosis can be requested through the study website https://internal.mesa-nhlbi.org/. Data for the HIV study may be requested from study collaborators: Lesley Inker, Lesley.inker@tuftsmedicine.org).

## Supplemental Material

Supplemental material is available at *Clinical Chemistry* online.

## Supplementary Material

hvaf110_Supplementary_Data

## Nonstandard Abbreviations

GFR, glomerular filtration rate; mGFR, measured glomerular filtration rate; eGFR, estimated glomerular filtration rate; eGFRcr, estimated glomerular filtration rate using creatinine; eGFRcys, estimated glomerular filtration rate using cystatin C; eGFRcr-cys, estimated glomerular filtration rate using creatinine and cystatin C; eGFRdiff, estimated glomerular filtration rate difference; HIV, human immunodeficiency virus; MESA, Multi-Ethnic Study of Atherosclerosis; BMI, body mass index; RMSE, root mean square error.

## The Multi-Ethnic Study of Atherosclerosis (MESA-Kidney) Study Group

**Collaborators**Tariq Shafi^a^, Wendy Post^b^, Peter Rossing^c^.

^a^Division of Nephrology, Baylor Scott and White Health, Temple, Texas, United States; ^b^Department of Epidemiology, Johns Hopkins University, Baltimore, Maryland, United States; ^c^Steno Diabetes Center Copenhagen, Herlev, Denmark.

## The HIV Study Group

**Collaborators**Christina Wyatt^a^, Zipporah Krishnasami^b^, James Hellinger^c^.

^a^Division of Nephrology, Department of Medicine, Duke University School of Medicine, Durham, North Carolina, United States; ^b^Division of Nephrology, Medical University of South Carolina, Charleston, South Carolina, United States; ^c^Division of Infectious Diseases, Tufts Medical Center, Boston, Massachusetts, United States.
