# Supplementary material for: Quantitative Analysis of a Novel Metabolite Panel to Estimate GFR (Panel eGFR) in Serum and Plasma Using LC-MS/MS
Source: Clin Chem. 2025 Oct 10;71(12):1269–80. doi: 10.1093/clinchem/hvaf110 (PMC12670587; doi:10.1093/clinchem/hvaf110)
Supplement: hvaf110_Supplementary_Data [file hvaf110_supplementary_data.docx]

Supplementary Material

Quantitative Analysis of a Novel Metabolite Panel to estimate GFR (panel eGFR) in Serum and Plasma using Liquid Chromatography Tandem Mass Spectrometry (LC-MS/MS)

Nora Fino, Lesley A. Inker, Seiei Shiba, Ogechi M. Adingwupu, Josef Coresh, Ben Haaland, Michael G. Shlipak, Andrew Levey, Jesse C. Seegmiller

Contents

[**Additional methods** 3](#_Toc203461820)

[Analytical survey 3](#_Toc203461821)

[Reagents and Instrumentation, Calibrator, Internal Standard, and Quality Control Preparation 3](#_Toc203461822)

[Reagents 3](#_Toc203461823)

[Creation of stock solution and calibrators 4](#_Toc203461824)

[Sample Preparation 4](#_Toc203461825)

[Table S1: Intermediate Stock Calibrator Formulation 5](#_Toc203461826)

[Table S2: Working Calibrator Formulation 6](#_Toc203461827)

[Table S3: Calibration Curve Standard Concentrations 7](#_Toc203461828)

[Table S4: Internal standard (IS) name, molecular weight, and concentration in working IS solution 8](#_Toc203461829)

[Creatinine and Cystatin C Analysis Details 9](#_Toc203461830)

[Table S5: Ionization Mode MRM transitions. 10](#_Toc203461831)

[**Supplemental Results** 11](#_Toc203461832)

[Table S6: Calibration Imprecision Data N = 21 Days 12](#_Toc203461833)

[Table S7: Linearity Studies 14](#_Toc203461834)

[Table S8: Recovery in SST Serum, RST Serum, EDTA Plasma and Lithium plasma 16](#_Toc203461835)

[Table S9: Agreement between pairwise sample types. 18](#_Toc203461836)

[Table S10: Agreement between pairwise sample types, after removing outliers. 19](#_Toc203461837)

[Table S11: Comparison of individuals randomly selected versus those not selected from the MESA-Kidney and HIV studies. 20](#_Toc203461838)

[Table S12: Comparison of the relationship of the markers with mGFR among untargeted and targeted assays. 21](#_Toc203461839)

[Figure S1: Creation of clinical validation dataset. 22](#_Toc203461840)

[Figure S2: Representative chromatograms for the lowest calibrator (S1) and a low specimen for each metabolite in a serum specimen. 23](#_Toc203461841)

[Figure S3: Impact of adding individual demographic terms to single marker panel 25](#_Toc203461842)

# **Additional methods**

## Analytical survey

From the set of metabolites identified from the global metabolomics study, we evaluated each metabolite along the analytical survey criteria. Those that survived this survey were thought to be excellent candidates for inclusion in the LC-MS/MS measurement procedure for a panel GFR estimate.

These factors included ion suppression/enhancement, retention time, chromatographic performance, dynamic range that was not consistent with others, matrix interference, and commercially availability of metabolites their isotopically labeled metabolites. For the metabolites that passed this initial examination, we then excluded metabolites with average percent difference between expected and experimental values for analyte greater than 15%; that failed specimen stability at 4, -20, and -80˚C for 7 days; or exhibited unstable working calibrators.

### Reagents and Instrumentation, Calibrator, Internal Standard, and Quality Control Preparation

### Reagents

The source of reagents are as follows: Water, acetonitrile, methanol, and 1x phosphate buffered saline (PBS) pH 7.4 (catalog# 10010-023) containing 1.06 mM monobasic potassium phosphate, 155.17 mM sodium chloride and 2.97 mM dibasic sodium phosphate were obtained from Thermo Fisher Scientific (Waltham, MA, USA). The water, acetonitrile and methanol were of high-performance liquid chromatography (HPLC) grade. Formic acid was obtained from RICCA Chemical (Arlington, TX, USA). 1-Methylimidazoleacetate, 3-hydroxy-3-methylglutarate, 4-acetamidobutyric acid, adipoyl-L-carnitine lithium salt, asymmetric dimethylarginine (ADMA), creatinine, D-(+)-arabitol, DL-4-hydroxy-3-methoxymandelic acid (VMA), D-xylonic acid lithium salt, D-erythronic acid potassium salt, D-gulonic acid lithium salt, N-acetyl-L-alanine, N^G^,N^G′^ -Dimethyl-L-arginine (SDMA) di(p-hydroxyazobenzene-p′-sulfonate) salt, adipoyl-L-carnitine-(N-methyl-d3) lithium salt, (+\-)-4-hydroxy-3-methoxymandelic acid (ring-D3) solution, ammonium hydroxide solution, and Bovine Serum Albumin lyophilized powder (BSA) were obtained from Sigma-Aldrich (St. Louis, MO, USA). Stripped serum used consisted of Mass Spect Gold Human Serum, Ultra-Low Vitamin D, Lipid Free from Golden West Diagnostics (catalog#MSG1000) (Temecula, CA, USA). 1-Methylhistidine, 1-methyl-Inosine, 2-(α-D-mannopyranosyl)-L-tryptophan, 3-methylglutaconic Acid, 3-methylglutarylcarnitine chloride, acetyl-L-threonine, kynurenic acid, L-homocitrulline, myo-inositol, N^2^,N^2^-dimethylguanosine, N6-carbamoylthreonyladenosine, N6-succinyl adenosine, N-acetylserine, N-formyl-L-methionine, N-methylguanidine hydrochloride, orotidine, pseudouridine, vanillactic acid, 1-methyl-Inosine-d3, 2-(dimethylamino)guanosine-d6, 2-(α-D-mannopyranosyl)-L-tryptophan-d4, 3-hydroxy-3-methyl-d3-pentanedioic acid, β-pseudouridine-^13^C, ^15^N2, D-arabinitol-^13^C5, myo-inositol-d6, N6-(N-threonylcarbonyl)adenosine-^13^C4,^15^N, N-acetyl-d3-L-threonine-2,3-d2, N-acetyl-L-serine-2,3,3-d3, N-acetylneuraminic acid-^13^C,d3, N^G^,N^G’^-dimethyl-L-arginine-d6, and vanillactic acid-^13^C3 were obtained from Toronto Research Chemicals (North York, ON, Canada). 3-Methylglutaconic acid-^13^C3, creatinine-d3, and τ-(methyl-d3)-L-histidine were obtained from Cambridge Isotope Laboratories, Inc. (Tewksbury, MA, USA). Guanidinosuccinic acid was obtained from Cayman Chemical (Ann Arbor, MI, USA).

Kynurenic-3,5,6,7,8-d5 acid, L-homocitrulline-2,6,6-d3, and N-acetyl-L-alanine-2,3,3,3-d4 were obtained from CDN Isotopes (Pointe-Claire, QC, Canada). N-Acetylneuraminic acid was obtained from Santa Cruz Biotechnology (Dallas, TX, USA). Vacufuge plus Centrifuge Concentrator was obtained from Eppendorf (Hamburg, Germany). VX-2500 Multi-Tube Vortexer was obtained from VWR (Radnor, PA, USA). MS104S Analytical Balance was obtained from Mettler Toledo (Columbus, OH, USA).

### Creation of stock solution and calibrators

All compounds were dissolved in 50% acetonitrile to create individual stock solutions at a concentration of 10 mg/mL for Pseudouridine and 1 mg/mL for all the other compounds. 50% acetonitrile with 0.1% ammonium hydroxide was used to create the kynurenic acid 1 mg/mL stock solution.

The intermediate calibrator stock solution was prepared by adding the appropriate volume of each stock solution provided in Table S1 into 5% w/v bovine serum albumin (BSA) in phosphate buffered saline (PBS). Nine calibrators were prepared by spiking the intermediate calibrator stock solution into 5% w/v BSA in PBS. The internal standard solution was created from 1 mg/mL of each isotopically labeled compound into 50% acetonitrile and the final internal standard solution was in 50% acetonitrile with the final concentrations listed in Table S4. Low, medium, and high-quality controls were prepared by adding 0.5 mL, 2 mL and 10 mL of the intermediate calibrator stock solution, respectively, into 50 mL class A volumetric flasks, followed by dilution with stripped serum to volume. All calibrators and quality controls were aliquoted as 0.40 mL and frozen at –80°C until used.

### Sample Preparation

The working calibrators, quality controls, and specimens were thawed at 25^o^C for one hour and mixed by inversion 10 times prior to preparation. All calibrators, controls, and specimens were processed by adding 100 µL to a 1.5 mL microcentrifuge tube (Sarstedt-Newton, North Carolina, USA) and 20 µL of internal standard solution followed by 400 µL acetonitrile with 0.1% formic acid. Resulting solutions were mixed using a multitube vortexer (VWR VX-2500, Radnor, Pennsylvania, USA) for 1 minute on setting 10 and then centrifuged (Eppendorf 5424, Hamburg, Germany) at 15,000 rcf for 2 minutes. A 200 µL aliquot of the supernatant was dried down using a Vacufuge plus Centrifuge Concentrator at 45°C for 2 hours and reconstituted in 200 µL of 5% acetonitrile with 0.1% formic acid, mixed by vortexing 60 seconds. This sample was then transferred into a 250 µL glass insert and placed into a 2 mL autosampler vial (Chrome Tech, Apple Valley, MN, USA) for analysis.

### Table S1: Intermediate Stock Calibrator Formulation

| **Compound name** | **Molecular weight (g/mol)** | **Purity** | **Concentration of individual stock solution (mg/mL)** | **Target concentration of intermediate stock solution (µM)** | **Volume of stock solution added to make 50 mL of intermediate stock solution (µL)** |
| --- | --- | --- | --- | --- | --- |
| N-Acetyl-L-serine | 147.13 | 0.90 | 1 | 75 | 613.0 |
| Pseudouridine | 244.2 | 0.95 | 10 | 125 | 160.7 |
| 2-(α-D-Mannopyranosyl)-tryptophan | 366.37 | 0.98 | 1 | 10 | 186.9 |
| N-Acetylneuraminic acid | 309.3 | 0.98 | 1 | 50 | 789.0 |
| 4-Acetamidobutanoic acid | 145.16 | 0.98 | 1 | 25 | 185.2 |
| Acetyl-L-threonine | 161.16 | 0.97 | 1 | 30 | 249.2 |
| N2, N2-dimethylguanosine | 311.29 | 0.98 | 1 | 3 | 47.6 |
| L-Homocitrulline | 189.21 | 0.96 | 1 | 25 | 246.4 |
| Kynurenic acid | 189.17 | 0.98 | 1 | 3 | 29.0 |
| N-Acetyl-L-alanine | 131.13 | 0.99 | 1 | 50 | 331.1 |
| SDMA | 758.82 | 0.99 | 1 | 25 | 958.1 |

* Diluent was 5% w/v BSA in PBS (1x)

### Table S2: Working Calibrator Formulation

| Table. Calibration scheme of working calibrators. | | |  |  |
| --- | --- | --- | --- | --- |
| **Calibrator name** | **Total volume (mL)** | **Stock solution added** | **Stock volume (mL)** | **Diluent volume (mL)*** |
| S1 | 20 | S5 | 1 | 19 |
| S2 | 20 | S7 | 0.4 | 19.6 |
| S3 | 20 | S7 | 1 | 19 |
| S4 | 20 | Intermediate stock solution | 0.4 | 19.6 |
| S5 | 20 | Intermediate stock solution | 0.8 | 19.2 |
| S6 | 20 | Intermediate stock solution | 2 | 18 |
| S7 | 20 | Intermediate stock solution | 4 | 16 |
| S8 | 20 | Intermediate stock solution | 6 | 14 |
| S9 | 20 | Intermediate stock solution | 8 | 12 |
| * Diluent was 5% w/v BSA in PBS (1x) | | |  |  |

### Table S3: Calibration Curve Standard Concentrations

| **Calibrator** | **S1** | **S2** | **S3** | **S4** | **S5** | **S6** | **S7** | **S8** | **S9** | **Intermediate Stock Standard** |
| --- | --- | --- | --- | --- | --- | --- | --- | --- | --- | --- |
| **N-Acetyl-L-serine (µM)** | 0.15 | 0.3 | 0.75 | 1.5 | 3 | 7.5 | 15 | 22.5 | 30 | 75 |
| **β-Pseudouridine (µM)** | 0.25 | 0.5 | 1.25 | 2.5 | 5 | 12.5 | 25 | 37.5 | 50 | 125 |
| **2-(α-D-Mannopyranosyl)-L-tryptophan (µM)** | 0.02 | 0.04 | 0.1 | 0.2 | 0.4 | 1 | 2 | 3 | 4 | 10 |
| **N-Acetylneuraminic acid (µM)** | 0.1 | 0.2 | 0.5 | 1 | 2 | 5 | 10 | 15 | 20 | 50 |
| **4-Acetamidobutanoic acid (µM)** | 0.05 | 0.1 | 0.25 | 0.5 | 1 | 2.5 | 5 | 7.5 | 10 | 25 |
| **N-Acetylthreonine (µM)** | 0.06 | 0.12 | 0.3 | 0.6 | 1.2 | 3 | 6 | 9 | 12 | 30 |
| **N^2^,N^2^-Dimethylguanosine (µM)** | 0.006 | 0.012 | 0.03 | 0.06 | 0.12 | 0.3 | 0.6 | 0.9 | 1.2 | 3 |
| **L-Homocitrulline (µM)** | 0.05 | 0.1 | 0.25 | 0.5 | 1 | 2.5 | 5 | 7.5 | 10 | 25 |
| **Kynurenic Acid (µM)** | 0.006 | 0.012 | 0.03 | 0.06 | 0.12 | 0.3 | 0.6 | 0.9 | 1.2 | 3 |
| **N-Acetyl-L-alanine (µM)** | 0.1 | 0.2 | 0.5 | 1 | 2 | 5 | 10 | 15 | 20 | 50 |
| **SDMA (µM)** | 0.05 | 0.1 | 0.25 | 0.5 | 1 | 2.5 | 5 | 7.5 | 10 | 25 |

### Table S4: Internal standard (IS) name, molecular weight, and concentration in working IS solution

| **IS Name** | **Molecular Weight (g/mol)** | **Concentration in working IS solution (µM)** |
| --- | --- | --- |
| N-Acetyl-L-serine-2,3,3-d3 | 150.15 | 25 |
| b-Pseudouridine-13C, 15N2 | 247.18 | 50 |
| 2-(α-D-Mannopyranosyl)-L-tryptophan-d4 | 370.39 | 2.5 |
| N^G^,N^G’^-Dimethyl-L-arginine-d6 | 208.29 | 10 |
| N-Acetylneuraminic Acid-^13^C,d3 | 313.28 | 25 |
| N-Acetyl-d3-L-threonine-2,3-d2 | 166.19 | 20 |
| 2-(Dimethylamino)guanosine-d6 | 317.33 | 2 |
| L-Homocitrulline-2,6,6-d3 | 192.23 | 5 |
| Kynurenic-3,5,6,7,8-d5 Acid | 194.20 | 2.5 |
| N-Acetyl-L-alanine-2,3,3,3-d4 | 135.16 | 20 |

### Creatinine and Cystatin C Analysis Details

In the HIV study, serum creatinine was measured on the Roche-Hitachi P-Module instrument with Roche Creatininase Plus assay (Hoffman-La Roche, Ltd., Basel, Switzerland) enzymatic method, traceable to Isotope Dilution Mass Spectroscopy (IDMS), and serum cystatin C was measured on the Siemens Pro-spec instrument particle-enhanced immuno-nephelometric assay, with calibration traceable to the ERM-DA47/IFCC reference material.(1, 2) In MESA-Kidney, serum creatinine was assayed by the Roche enzymatic method on a Roche Modular P Chemistry Analyzer (Roche Diagnostics Corporation) (CV 2.3%). The method is calibrated using a standard traceable to IDMS. Serum cystatin C was assayed by the Gentian turbidimetric method (Gentian AS, Moss, Norway) on a Roche COBAS 6000 chemistry analyzer (Roche Diagnostics) (CV 4.3% at 0.75 mg/L and 3.2% at 3.83 mg/L). The Gentian cystatin C calibration was traceable to ERM-DA47/IFCC reference material.

### Table S5: Ionization Mode MRM transitions.

| **Ionization mode** | **Quant/Qual** | **Analyte name** | **Q1 / Q3** | **IS name** | **IS Q1 / Q3** |
| --- | --- | --- | --- | --- | --- |
| Positive | Quant | Pseudouridine | 245.1 / 191.0 | β-Pseudouridine-13C, 15N2 | 248.1 / 194.0 |
| Positive | Qual | Pseudouridine | 245.1 / 209.0 | β-Pseudouridine-13C, 15N2 | 248.1 / 212.0 |
| Positive | Quant | 2-(α-D-Mannopyranosyl)-tryptophan | 367.1 / 156.1 | 2-(alpha-D-Mannopyranosyl)-tryptophan-d4 | 371.1 / 160.1 |
| Positive | Qual | 2-(α-D-Mannopyranosyl)-tryptophan | 367.1 / 247.1 | 2-(alpha-D-Mannopyranosyl)-tryptophan-d4 | 371.1 / 251.1 |
| Positive | Quant | SDMA | 203.1 / 172.1 | SDMA-d6 | 209.1 / 175.2 |
| Positive | Quant | L-Homocitrulline | 190.1 / 173.1 | L-Homocitrulline-2,6,6-d3 | 193.1 / 176.1 |
| Positive | Qual | L-Homocitrulline | 190.1 / 127.1 | L-Homocitrulline-2,6,6-d3 | 193.1 / 130.1 |
| Positive | Quant | Kynurenic acid | 190.0 / 89.1 | Kynurenic acid-3,5,6,7,8-d5 | 195.0 / 94.1 |
| Positive | Qual | Kynurenic acid | 190.0 / 116.2 | Kynurenic acid-3,5,6,7,8-d5 | 195.0 / 121.2 |
| Positive | Quant | N2,N2-dimethylguanosine | 312.1 / 180.0 | N2,N2-dimethylguanosine-d6 | 318.1 / 186.0 |
| Positive | Qual | N2,N2-dimethylguanosine | 312.1 / 135.0 | N2,N2-dimethylguanosine-d6 | 318.1 / 135.0 |
| Negative | Quant | N-Acetylserine | 146.1 / 116.0 | N-Acetylserine-2,3,3-d3 | 149.1 / 117.0 |
| Negative | Qual | N-Acetylserine | 146.1 / 74.0 | N-Acetylserine-2,3,3-d3 | 149.1 / 75.0 |
| Negative | Quant | N-Acetylneuraminic acid | 308.1 / 98.0 | N-Acetylneuraminic acid-13C,d3 | 312.1 / 102.0 |
| Negative | Qual | N-Acetylneuraminic acid | 308.1 / 170.0 | N-Acetylneuraminic acid-13C,d3 | 312.1 / 174.0 |
| Negative | Quant | Acetyl-L-threonine | 160.1 / 74.0 | N-Acetyl-d3-L-threonine-2,3-d2 | 165.1 / 76.0 |
| Negative | Qual | Acetyl-L-threonine | 160.1 / 116.0 | N-Acetyl-d3-L-threonine-2,3-d2 | 165.1 / 120.0 |
| Negative | Quant | N-Acetyl-L-alanine | 130.1 / 88.0 | N-acetyl-L-alanine-d4 | 134.1 / 92.0 |
| Negative | Quant | 4-Acetamidobutanoic acid | 144.1 / 102.0 | N-acetyl-L-alanine-d4 | 134.1 / 92.0 |
| Negative | Qual | 4-Acetamidobutanoic acid | 144.1 / 100.0 | N-acetyl-L-alanine-d4 | 134.1 / 92.0 |

# **Supplemental Results**

## Table S6: Calibration Imprecision Data N = 21 Days

| **Metabolite** | **Calibration Conc (µM)** | Average (µM) | Std Dev (µM) | %CV |  | **Metabolite** | **Calibration Conc (µM)** | Average (µM) | Std Dev (µM) | %CV |
| --- | --- | --- | --- | --- | --- | --- | --- | --- | --- | --- |
| **Pseudouridine** | **0.25** | 0.26 | 0.0060 | 2.35% |  | **N-Acetylserine** | **0.15** | 0.15 | 0.0068 | 4.38% |
|  | **0.5** | 0.48 | 0.0071 | 1.46% |  |  | **0.3** | 0.29 | 0.0077 | 2.63% |
|  | **1.25** | 1.26 | 0.0187 | 1.49% |  |  | **0.75** | 0.75 | 0.0202 | 2.69% |
|  | **2.5** | 2.45 | 0.0303 | 1.24% |  |  | **1.5** | 1.47 | 0.0259 | 1.76% |
|  | **5** | 5.05 | 0.0568 | 1.12% |  |  | **3** | 3.02 | 0.0432 | 1.43% |
|  | **12.5** | 12.73 | 0.1294 | 1.02% |  |  | **7.5** | 7.62 | 0.1071 | 1.41% |
|  | **25** | 24.79 | 0.2531 | 1.02% |  |  | **15** | 14.93 | 0.2107 | 1.41% |
|  | **37.5** | 37.45 | 0.2510 | 0.67% |  |  | **22.5** | 22.42 | 0.2442 | 1.09% |
|  | **50** | 50.03 | 0.3886 | 0.78% |  |  | **30** | 30.05 | 0.3435 | 1.14% |
| **2-(α-D-Mannopyranosyl)-tryptophan** | **0.02** | 0.02 | 0.0018 | 8.58% |  | **N-Acetylneuraminic acid** | **0.1** | 0.10 | 0.0077 | 7.75% |
|  | **0.04** | 0.04 | 0.0030 | 7.61% |  |  | **0.2** | 0.19 | 0.0152 | 7.81% |
|  | **0.1** | 0.10 | 0.0048 | 4.83% |  |  | **0.5** | 0.51 | 0.0255 | 5.03% |
|  | **0.2** | 0.19 | 0.0112 | 5.78% |  |  | **1** | 0.98 | 0.0355 | 3.62% |
|  | **0.4** | 0.40 | 0.0224 | 5.65% |  |  | **2** | 2.05 | 0.0757 | 3.69% |
|  | **1** | 1.02 | 0.0589 | 5.79% |  |  | **5** | 5.10 | 0.1376 | 2.70% |
|  | **2** | 1.96 | 0.0662 | 3.37% |  |  | **10** | 9.98 | 0.1291 | 1.29% |
|  | **3** | 2.98 | 0.0987 | 3.32% |  |  | **15** | 14.99 | 0.2317 | 1.55% |
|  | **4** | 4.05 | 0.0955 | 2.36% |  |  | **20** | 19.90 | 0.2634 | 1.32% |
| **SDMA** | **0.05** | 0.05 | 0.0014 | 2.60% |  | **Acetyl-L-threonine** | **0.06** | 0.06 | 0.0016 | 2.59% |
|  | **0.1** | 0.10 | 0.0025 | 2.60% |  |  | **0.12** | 0.12 | 0.0021 | 1.80% |
|  | **0.25** | 0.25 | 0.0051 | 2.06% |  |  | **0.3** | 0.30 | 0.0037 | 1.24% |
|  | **0.5** | 0.49 | 0.0085 | 1.74% |  |  | **0.6** | 0.59 | 0.0069 | 1.16% |
|  | **1** | 1.00 | 0.0215 | 2.15% |  |  | **1.2** | 1.21 | 0.0168 | 1.38% |
|  | **2.5** | 2.51 | 0.0629 | 2.51% |  |  | **3** | 3.06 | 0.0452 | 1.48% |
|  | **5** | 4.99 | 0.0534 | 1.07% |  |  | **6** | 5.96 | 0.0613 | 1.03% |
|  | **7.5** | 7.49 | 0.0499 | 0.67% |  |  | **9** | 8.98 | 0.0746 | 0.83% |
|  | **10** | 10.02 | 0.0708 | 0.71% |  |  | **12** | 12.00 | 0.0954 | 0.80% |
| **L-Homocitrulline** | **0.05** | 0.05 | 0.0020 | 3.83% |  | **N-Acetyl-L-alanine** | **0.1** | 0.10 | 0.0023 | 2.22% |
|  | **0.1** | 0.10 | 0.0027 | 2.82% |  |  | **0.2** | 0.19 | 0.0029 | 1.51% |
|  | **0.25** | 0.25 | 0.0037 | 1.46% |  |  | **0.5** | 0.50 | 0.0053 | 1.05% |
|  | **0.5** | 0.49 | 0.0086 | 1.75% |  |  | **1** | 0.99 | 0.0135 | 1.36% |
|  | **1** | 1.02 | 0.0162 | 1.59% |  |  | **2** | 2.02 | 0.0216 | 1.07% |
|  | **2.5** | 2.56 | 0.0345 | 1.35% |  |  | **5** | 5.10 | 0.0420 | 0.82% |
|  | **5** | 4.98 | 0.0688 | 1.38% |  |  | **10** | 9.96 | 0.0802 | 0.81% |
|  | **7.5** | 7.49 | 0.0967 | 1.29% |  |  | **15** | 14.96 | 0.1140 | 0.76% |
|  | **10** | 9.96 | 0.0875 | 0.88% |  |  | **20** | 19.96 | 0.1556 | 0.78% |

| **Kynurenic acid** | **0.006** | 0.006 | 0.0003 | 5.37% |  | **4-Acetamidobutanoic acid** | **0.05** | 0.05 | 0.0014 | 2.64% |
| --- | --- | --- | --- | --- | --- | --- | --- | --- | --- | --- |
|  | **0.012** | 0.012 | 0.0005 | 4.47% |  |  | **0.1** | 0.10 | 0.0027 | 2.72% |
|  | **0.03** | 0.030 | 0.0010 | 3.24% |  |  | **0.25** | 0.25 | 0.0037 | 1.50% |
|  | **0.06** | 0.059 | 0.0012 | 2.02% |  |  | **0.5** | 0.49 | 0.0115 | 2.36% |
|  | **0.12** | 0.121 | 0.0021 | 1.70% |  |  | **1** | 0.99 | 0.0140 | 1.41% |
|  | **0.3** | 0.306 | 0.0041 | 1.34% |  |  | **2.5** | 2.52 | 0.0341 | 1.36% |
|  | **0.6** | 0.595 | 0.0063 | 1.06% |  |  | **5** | 4.94 | 0.0622 | 1.26% |
|  | **0.9** | 0.901 | 0.0083 | 0.92% |  |  | **7.5** | 7.50 | 0.0901 | 1.20% |
|  | **1.2** | 1.198 | 0.0086 | 0.72% |  |  | **10** | 10.07 | 0.1025 | 1.02% |
| **N2,N2-dimethylguanosine** | **0.006** | 0.007 | 0.0003 | 4.73% |  |  |  |  |  |  |
|  | **0.012** | 0.012 | 0.0004 | 3.82% |  |  |  |  |  |  |
|  | **0.03** | 0.030 | 0.0013 | 4.35% |  |  |  |  |  |  |
|  | **0.06** | 0.057 | 0.0023 | 4.03% |  |  |  |  |  |  |
|  | **0.12** | 0.119 | 0.0039 | 3.25% |  |  |  |  |  |  |
|  | **0.3** | 0.296 | 0.0137 | 4.62% |  |  |  |  |  |  |
|  | **0.6** | 0.595 | 0.0189 | 3.17% |  |  |  |  |  |  |
|  | **0.9** | 0.915 | 0.0260 | 2.85% |  |  |  |  |  |  |
|  | **1.2** | 1.198 | 0.0176 | 1.47% |  |  |  |  |  |  |

## Table S7: Linearity Studies

| **Metabolite** | **Obs, Exp, % diff** | **Dilution Factor** | | | | | | | | | **Stripped Serum Blank (μM)** | **Slope** | **Intercept** | **R^2^** | **Ave % Difference** |
| --- | --- | --- | --- | --- | --- | --- | --- | --- | --- | --- | --- | --- | --- | --- | --- |
|  |  | **0** | **x2** | **x4** | **x8** | **x16** | **x32** | **x64** | **x128** | **x256** |  |  |  |  |  |
| Pseudouridine | O | 49.59 | 25.30 | 13.06 | 6.49 | 3.25 | 1.67 | 0.82 | 0.41 | 0.19 | < 0 | 1.0015 | 0.185 | 0.99979 | 4.73% |
|  | E | 49.59 | 24.79 | 12.40 | 6.20 | 3.10 | 1.55 | 0.77 | 0.39 | 0.19 |  |  |  |  |  |
|  | % | 0.00% | 2.03% | 5.35% | 4.78% | 4.90% | 7.97% | 6.28% | 6.53% | 0.00% |  |  |  |  |  |
| 2-(alpha-D-Mannopyranosyl)-tryptophan | O | 4.22 | 2.15 | 1.06 | 0.56 | 0.24 | 0.14 | 0.07 | 0.04 | 0.02 | < 0 | 1.0026 | 0.005 | 0.99983 | 3.00% |
|  | E | 4.22 | 2.11 | 1.06 | 0.53 | 0.26 | 0.13 | 0.07 | 0.03 | 0.02 |  |  |  |  |  |
|  | % | 0.00% | 2.06% | 0.30% | 5.80% | -8.18% | 5.88% | 5.04% | 11.86% | 1.25% |  |  |  |  |  |
| SDMA | O | 10.36 | 5.24 | 2.74 | 1.37 | 0.70 | 0.35 | 0.18 | 0.09 | 0.05 | < 0 | 0.9991 | 0.046 | 0.99982 | 8.77% |
|  | E | 10.36 | 5.18 | 2.59 | 1.30 | 0.65 | 0.32 | 0.16 | 0.08 | 0.04 |  |  |  |  |  |
|  | % | 0.00% | 1.13% | 5.69% | 6.02% | 7.42% | 9.58% | 11.53% | 12.89% | 15.85% |  |  |  |  |  |
| L-Homocitrulline | O | 9.73 | 4.85 | 2.50 | 1.24 | 0.63 | 0.32 | 0.16 | 0.08 | 0.04 | < 0 | 0.9984 | 0.017 | 0.99994 | 2.94% |
|  | E | 9.73 | 4.86 | 2.43 | 1.22 | 0.61 | 0.30 | 0.15 | 0.08 | 0.04 |  |  |  |  |  |
|  | % | 0.00% | -0.39% | 2.93% | 1.85% | 4.13% | 4.71% | 4.74% | 2.37% | 3.16% |  |  |  |  |  |
| Kynurenic acid | O | 1.208 | 0.596 | 0.311 | 0.152 | 0.079 | 0.038 | 0.019 | 0.010 | 0.004 | < 0 | 0.9976 | 0.001 | 0.99989 | 0.27% |
|  | E | 1.208 | 0.604 | 0.302 | 0.151 | 0.076 | 0.038 | 0.019 | 0.009 | 0.005 |  |  |  |  |  |
|  | % | 0.00% | -1.29% | 2.95% | 0.40% | 4.11% | 1.46% | 1.72% | 1.72% | -8.87% |  |  |  |  |  |
| N2,N2-dimethylguanosine | O | 1.224 | 0.576 | 0.312 | 0.152 | 0.080 | 0.041 | 0.020 | 0.011 | 0.006 | 0.0007 | 0.9901 | 0.000 | 0.99913 | 3.16% |
|  | E | 1.224 | 0.612 | 0.307 | 0.154 | 0.077 | 0.039 | 0.020 | 0.010 | 0.005 |  |  |  |  |  |
|  | % | 0.00% | -5.88% | 1.83% | -1.30% | 3.43% | 5.33% | 0.44% | 8.23% | 13.18% |  |  |  |  |  |
| N-Acetylserine | O | 29.73 | 15.25 | 8.00 | 3.84 | 1.97 | 1.03 | 0.53 | 0.25 | 0.13 | < 0 | 1.0019 | 0.142 | 0.99963 | 8.71% |
|  | E | 29.73 | 14.86 | 7.43 | 3.72 | 1.86 | 0.93 | 0.46 | 0.23 | 0.12 |  |  |  |  |  |
|  | % | 0.00% | 2.57% | 7.65% | 3.34% | 5.96% | 10.86% | 14.52% | 9.03% | 15.74% |  |  |  |  |  |
| N-Acetylneuraminic acid | O | 19.39 | 9.77 | 4.99 | 2.43 | 1.31 | 0.64 | 0.31 | 0.16 | 0.06 | < 0 | 0.9999 | 0.041 | 0.99993 | 1.46% |
|  | E | 19.39 | 9.70 | 4.85 | 2.42 | 1.21 | 0.61 | 0.30 | 0.15 | 0.08 |  |  |  |  |  |
|  | % | 0.00% | 0.72% | 2.99% | 0.24% | 8.33% | 6.01% | 2.97% | 6.60% | -16.17% |  |  |  |  |  |
| Acetyl-L-threonine | O | 11.85 | 6.08 | 3.12 | 1.55 | 0.80 | 0.40 | 0.20 | 0.10 | 0.05 | < 0 | 1.0020 | 0.049 | 0.99976 | 5.51% |
|  | E | 11.85 | 5.93 | 2.96 | 1.48 | 0.74 | 0.37 | 0.19 | 0.09 | 0.05 |  |  |  |  |  |
|  | % | 0.00% | 2.62% | 5.26% | 4.51% | 7.51% | 8.40% | 9.57% | 6.60% | -0.42% |  |  |  |  |  |
| N-Acetyl-L-alanine | O | 20.15 | 10.13 | 5.26 | 2.65 | 1.37 | 0.69 | 0.35 | 0.17 | 0.08 | < 0 | 0.9983 | 0.077 | 0.99989 | 6.86% |
|  | E | 20.15 | 10.08 | 5.04 | 2.52 | 1.26 | 0.63 | 0.31 | 0.16 | 0.08 |  |  |  |  |  |
|  | % | 0.00% | 0.54% | 4.44% | 5.14% | 8.43% | 9.13% | 10.36% | 9.25% | 7.60% |  |  |  |  |  |
| 4-Acetamidobutanoic acid | O | 10.49 | 5.22 | 2.68 | 1.34 | 0.71 | 0.35 | 0.18 | 0.09 | 0.04 | < 0 | 0.9971 | 0.026 | 0.99995 | 5.69% |
|  | E | 10.49 | 5.24 | 2.62 | 1.31 | 0.66 | 0.33 | 0.16 | 0.08 | 0.04 |  |  |  |  |  |
|  | % | 0.00% | -0.50% | 2.31% | 2.25% | 8.24% | 8.08% | 8.69% | 8.51% | 7.90% |  |  |  |  |  |

Units are μM, O, observed, E, expected; % Difference. Serial dilutions performed using stripped serum as diluent.

## Table S8: Recovery in SST Serum, RST Serum, EDTA Plasma and Lithium plasma

| **Spike Recovery Serum Separator Tube (SST)** | | | | | | | | | | | | | | | | | | | | | | | | | | | | | | | | | | |
| --- | --- | --- | --- | --- | --- | --- | --- | --- | --- | --- | --- | --- | --- | --- | --- | --- | --- | --- | --- | --- | --- | --- | --- | --- | --- | --- | --- | --- | --- | --- | --- | --- | --- | --- |
|  | | Sample (µM) | | Spike Low (µM) | | | Spike Low Result (µM) | | | Spike Low Recovery | | | Spike Med (µM) | | | Spike Med Result (µM) | | | Spike Med Recovery | | | Spike High (µM) | | | Spike High (µM) | | | Spike High Recovery | | | Mean Recovery | | | |
| **Pseudouridine** | | 3.19 | | 5.95 | | | 9.54 | | | 107% | | | 8.72 | | | 12.60 | | | 108% | | | 11.36 | | | 15.67 | | | 110% | | | 108% | | | |
| **2-(alpha-D-Mannopyranosyl)-tryptophan** | | 0.17 | | 0.48 | | | 0.70 | | | 110% | | | 0.70 | | | 0.92 | | | 107% | | | 0.91 | | | 1.20 | | | 113% | | | 110% | | | |
| **SDMA** | | 0.50 | | 1.19 | | | 1.76 | | | 106% | | | 1.74 | | | 2.30 | | | 103% | | | 2.27 | | | 3.03 | | | 112% | | | 107% | | | |
| **L-Homocitrulline** | | 0.16 | | 1.19 | | | 1.46 | | | 109% | | | 1.74 | | | 1.93 | | | 101% | | | 2.27 | | | 2.57 | | | 106% | | | 106% | | | |
| **Kynurenic acid** | | 0.053 | | 0.143 | | | 0.211 | | | 110% | | | 0.209 | | | 0.274 | | | 106% | | | 0.273 | | | 0.350 | | | 109% | | | 108% | | | |
| **N2,N2-dimethylguanosine** | | 0.033 | | 0.143 | | | 0.186 | | | 107% | | | 0.209 | | | 0.258 | | | 107% | | | 0.273 | | | 0.329 | | | 109% | | | 108% | | | |
| **N-Acetylserine** | | 0.94 | | 3.57 | | | 4.86 | | | 110% | | | 5.23 | | | 6.48 | | | 106% | | | 6.82 | | | 8.54 | | | 112% | | | 109% | | | |
| **N-Acetylneuraminic acid** | | 0.68 | | 2.38 | | | 3.21 | | | 106% | | | 3.49 | | | 4.37 | | | 106% | | | 4.55 | | | 5.47 | | | 105% | | | 106% | | | |
| **Acetyl-L-threonine** | | 0.45 | | 1.43 | | | 2.00 | | | 109% | | | 2.09 | | | 2.61 | | | 103% | | | 2.73 | | | 3.38 | | | 108% | | | 107% | | | |
| **N-Acetyl-L-alanine** | | 1.32 | | 2.38 | | | 3.79 | | | 104% | | | 3.49 | | | 4.93 | | | 103% | | | 4.55 | | | 6.16 | | | 106% | | | 104% | | | |
| **4-Acetamidobutanoic acid** | | 0.17 | | 1.19 | | | 1.37 | | | 101% | | | 1.74 | | | 1.92 | | | 101% | | | 2.27 | | | 2.51 | | | 103% | | | 102% | | | |
| **Spike Recovery Serum Red No Gel Tube (RST)** | | | | | | | | | | | | | | | | | | | | | | | | | | | | | | | | | | |
|  | Sample (µM) | | | | | Spike Low (µM) | | | Spike Low Result (µM) | | | Spike Low Recovery | | | Spike Med (µM) | | | Spike Med Result (µM) | | | Spike Med Recovery | | | Spike High (µM) | | | Spike High (µM) | | | Spike High Recovery | | | Mean Recovery | |
| **Pseudouridine** | 3.19 | | | | | 5.95 | | | 9.05 | | | 98% | | | 8.72 | | | 12.45 | | | 106% | | | 11.36 | | | 15.56 | | | 109% | | | 104% | |
| **2-(alpha-D-Mannopyranosyl)-tryptophan** | 0.17 | | | | | 0.48 | | | 0.70 | | | 110% | | | 0.70 | | | 0.95 | | | 111% | | | 0.91 | | | 1.11 | | | 104% | | | 108% | |
| **SDMA** | 0.48 | | | | | 1.19 | | | 1.65 | | | 98% | | | 1.74 | | | 2.33 | | | 106% | | | 2.27 | | | 3.03 | | | 112% | | | 105% | |
| **L-Homocitrulline** | 0.16 | | | | | 1.19 | | | 1.32 | | | 97% | | | 1.74 | | | 1.99 | | | 105% | | | 2.27 | | | 2.58 | | | 107% | | | 103% | |
| **Kynurenic acid** | 0.054 | | | | | 0.143 | | | 0.193 | | | 97% | | | 0.209 | | | 0.276 | | | 106% | | | 0.273 | | | 0.344 | | | 106% | | | 103% | |
| **N2,N2-dimethylguanosine** | 0.031 | | | | | 0.143 | | | 0.176 | | | 102% | | | 0.209 | | | 0.257 | | | 108% | | | 0.273 | | | 0.337 | | | 112% | | | 107% | |
| **N-Acetylserine** | 0.94 | | | | | 3.57 | | | 4.49 | | | 99% | | | 5.23 | | | 6.46 | | | 106% | | | 6.82 | | | 8.29 | | | 108% | | | 104% | |
| **N-Acetylneuraminic acid** | 0.69 | | | | | 2.38 | | | 2.80 | | | 89% | | | 3.49 | | | 4.05 | | | 96% | | | 4.55 | | | 5.31 | | | 102% | | | 96% | |
| **Acetyl-L-threonine** | 0.51 | | | | | 1.43 | | | 1.84 | | | 94% | | | 2.09 | | | 2.58 | | | 99% | | | 2.73 | | | 3.38 | | | 105% | | | 99% | |
| **N-Acetyl-L-alanine** | 1.30 | | | | | 2.38 | | | 3.59 | | | 96% | | | 3.49 | | | 4.93 | | | 104% | | | 4.55 | | | 6.12 | | | 106% | | | 102% | |
| **4-Acetamidobutanoic acid** | 0.16 | | | | | 1.19 | | | 1.27 | | | 93% | | | 1.74 | | | 1.91 | | | 101% | | | 2.27 | | | 2.53 | | | 104% | | | 99% | |
| **Spike Recovery EDTA Plasma (EDTA)** | | | | | | | | | | | | | | | | | | | | | | | | | | | | | | | | | |  |
|  | | | Sample (µM) | | Spike Low (µM) | | | Spike Low Result (µM) | | | Spike Low Recovery | | | Spike Med (µM) | | | Spike Med Result (µM) | | | Spike Med Recovery | | | Spike High (µM) | | | Spike High (µM) | | | Spike High Recovery | | | Mean Recovery | |  |
| **Pseudouridine** | | | 3.13 | | 5.95 | | | 9.49 | | | 107% | | | 8.72 | | | 12.44 | | | 107% | | | 11.36 | | | 15.26 | | | 107% | | | 107% | |  |
| **2-(alpha-D-Mannopyranosyl)-tryptophan** | | | 0.16 | | 0.48 | | | 0.68 | | | 108% | | | 0.70 | | | 0.92 | | | 108% | | | 0.91 | | | 1.14 | | | 108% | | | 108% | |  |
| **SDMA** | | | 0.51 | | 1.19 | | | 1.75 | | | 104% | | | 1.74 | | | 2.53 | | | 116% | | | 2.27 | | | 3.01 | | | 110% | | | 110% | |  |
| **L-Homocitrulline** | | | 0.17 | | 1.19 | | | 1.45 | | | 108% | | | 1.74 | | | 2.08 | | | 110% | | | 2.27 | | | 2.68 | | | 111% | | | 109% | |  |
| **Kynurenic acid** | | | 0.055 | | 0.143 | | | 0.207 | | | 107% | | | 0.209 | | | 0.283 | | | 109% | | | 0.273 | | | 0.352 | | | 109% | | | 108% | |  |
| **N2,N2-dimethylguanosine** | | | 0.031 | | 0.143 | | | 0.193 | | | 113% | | | 0.209 | | | 0.266 | | | 112% | | | 0.273 | | | 0.325 | | | 108% | | | 111% | |  |
| **N-Acetylserine** | | | 0.85 | | 3.57 | | | 4.67 | | | 107% | | | 5.23 | | | 6.40 | | | 106% | | | 6.82 | | | 8.11 | | | 106% | | | 106% | |  |
| **N-Acetylneuraminic acid** | | | 0.48 | | 2.38 | | | 2.92 | | | 103% | | | 3.49 | | | 4.05 | | | 103% | | | 4.55 | | | 5.39 | | | 108% | | | 104% | |  |
| **Acetyl-L-threonine** | | | 0.49 | | 1.43 | | | 1.88 | | | 98% | | | 2.09 | | | 2.68 | | | 105% | | | 2.73 | | | 3.27 | | | 102% | | | 102% | |  |
| **N-Acetyl-L-alanine** | | | 1.25 | | 2.38 | | | 3.65 | | | 101% | | | 3.49 | | | 4.87 | | | 104% | | | 4.55 | | | 6.04 | | | 105% | | | 103% | |  |
| **4-Acetamidobutanoic acid** | | | 0.16 | | 1.19 | | | 1.32 | | | 97% | | | 1.74 | | | 1.91 | | | 100% | | | 2.27 | | | 2.44 | | | 100% | | | 99% | |  |
| **Spike Recovery Lithium Heparin Plasma (HP)** | | | | | | | | | | | | | | | | | | | | | | | | | | | | | | | | | |  |
|  | | | Sample (µM) | Spike Low (µM) | | | Spike Low Result (µM) | | | Spike Low Recovery | | | Spike Med (µM) | | | Spike Med Result (µM) | | | Spike Med Recovery | | | Spike High (µM) | | | Spike High (µM) | | | Spike High Recovery | | | Mean Recovery | | |  |
| **Pseudouridine** | | | 3.13 | 5.95 | | | 9.37 | | | 105% | | | 8.72 | | | 12.14 | | | 103% | | | 11.36 | | | 15.44 | | | 108% | | | 105% | | |  |
| **2-(alpha-D-Mannopyranosyl)-tryptophan** | | | 0.15 | 0.48 | | | 0.64 | | | 104% | | | 0.70 | | | 0.90 | | | 108% | | | 0.91 | | | 1.12 | | | 107% | | | 106% | | |  |
| **SDMA 1** | | | 0.47 | 1.19 | | | 1.74 | | | 106% | | | 1.74 | | | 2.29 | | | 104% | | | 2.27 | | | 2.99 | | | 111% | | | 107% | | |  |
| **L-Homocitrulline** | | | 0.16 | 1.19 | | | 1.37 | | | 102% | | | 1.74 | | | 1.87 | | | 98% | | | 2.27 | | | 2.67 | | | 110% | | | 103% | | |  |
| **Kynurenic acid** | | | 0.053 | 0.143 | | | 0.205 | | | 106% | | | 0.209 | | | 0.266 | | | 102% | | | 0.273 | | | 0.351 | | | 109% | | | 106% | | |  |
| **N2,N2-dimethylguanosine** | | | 0.033 | 0.143 | | | 0.182 | | | 105% | | | 0.209 | | | 0.254 | | | 105% | | | 0.273 | | | 0.333 | | | 110% | | | 107% | | |  |
| **N-Acetylserine** | | | 0.91 | 3.57 | | | 4.69 | | | 106% | | | 5.23 | | | 6.40 | | | 105% | | | 6.82 | | | 8.50 | | | 111% | | | 107% | | |  |
| **N-Acetylneuraminic acid** | | | 0.58 | 2.38 | | | 2.91 | | | 98% | | | 3.49 | | | 3.82 | | | 93% | | | 4.55 | | | 5.53 | | | 109% | | | 100% | | |  |
| **Acetyl-L-threonine** | | | 0.46 | 1.43 | | | 1.90 | | | 101% | | | 2.09 | | | 2.61 | | | 103% | | | 2.73 | | | 3.39 | | | 107% | | | 104% | | |  |
| **N-Acetyl-L-alanine** | | | 1.27 | 2.38 | | | 3.67 | | | 101% | | | 3.49 | | | 4.78 | | | 101% | | | 4.55 | | | 6.11 | | | 107% | | | 103% | | |  |
| **4-Acetamidobutanoic acid** | | | 0.16 | 1.19 | | | 1.32 | | | 97% | | | 1.74 | | | 1.87 | | | 98% | | | 2.27 | | | 2.53 | | | 104% | | | 100% | | |  |

## Table S9: Agreement between pairwise sample types.

Using Deming regression, we estimated intercepts and slopes with their 95% confidence intervals. For each metabolite, we modeled the heparin‐plasma measurement as the dependent variable and the EDTA‐plasma measurement as the independent variable, and we ran analogous models comparing heparin plasma to serum and EDTA plasma to serum. Intercepts close to zero and slopes close to one indicate good agreement. The coefficient of variation (CV %) was calculated by computing each sample’s mean and standard deviation across measurement types to obtain a per‐sample CV (SD/mean), then averaging these per‐sample CVs.

|  | **Heparin Plasma ~ EDTA Plasma** | | | **Heparin Plasma ~ Serum** | | | **EDTA Plasma ~ Serum** | | | |
| --- | --- | --- | --- | --- | --- | --- | --- | --- | --- | --- |
|  | **Intercept (95% CI)** | **Slope(95% CI)** | **CV%** | **Intercept (95% CI)** | **Slope( 95% CI)** | **CV%** | **Intercept (95% CI)** | **Slope (95% CI)** | **CV%** |  |
| Pseudouridine | 0.04  (-0.041, 0.12) | 0.99  (0.97, 1) | 1.0% | -0.0099  (-0.12, 0.099) | 1  (0.97, 1) | 1.2% | -0.043  (-0.13, 0.047) | 1  (0.98, 1) | 1.0% |  |
| 2-(alpha-D-Mannopyranosyl)-tryptophan | 0.0031  (-0.008, 0.014) | 0.99  (0.95, 1) | 2.7% | 0.0033  (-0.0084, 0.015) | 0.94  (0.89, 0.98) | 4.7% | 0.0031  (-0.0084, 0.015) | 0.93  (0.88, 0.98) | 4.9% |  |
| SDMA | -0.0037  (-0.031, 0.024) | 1  (0.97, 1.1) | 1.8% | -0.00015  (-0.023, 0.023) | 0.99  (0.95, 1) | 1.7% | 0.0035  (-0.019, 0.026) | 0.97  (0.93, 1) | 2.2% |  |
| L-Homocitrulline | 0.0056  (-0.0046, 0.016) | 1  (0.97, 1) | 2.3% | -0.0021  (-0.013, 0.0085) | 1  (0.98, 1) | 2.1% | -0.0082  (-0.02, 0.0032) | 1  (0.97, 1) | 2.4% |  |
| Kynurenic acid | -0.00028  (-0.00098, 0.00042) | 1  (1, 1) | 2.1% | 0.00019  (-0.00069, 0.0011) | 0.98  (0.96, 0.99) | 2.9% | 0.00045  (-0.00045, 0.0014) | 0.96  (0.95, 0.98) | 3.1% |  |
| N2,N2-dimethylguanosine | 0.00053  (-0.0015, 0.0026) | 1  (0.97, 1.1) | 2.4% | 0.00012  (-0.0016, 0.0018) | 0.99  (0.95, 1) | 2.5% | -0.00043  (-0.0023, 0.0015) | 0.98  (0.93, 1) | 3.0% |  |
| N-Acetylserine | -0.0069  (-0.075, 0.061) | 1  (0.97, 1.1) | 2.3% | 0.024  (0.0044, 0.043) | 0.97  (0.96, 0.99) | 2.0% | 0.031  (-0.025, 0.086) | 0.95  (0.91, 0.99) | 2.5% |  |
| N-Acetylneuraminic acid | 0.11  (-0.032, 0.26) | 0.87  (0.68, 1.1) | 7.0% | -0.022  (-0.15, 0.1) | 1  (0.86, 1.2) | 5.5% | -0.16  (-0.26, -0.052) | 1.2  (1, 1.3) | 8.8% |  |
| Acetyl-L-threonine | 0.015  (-0.017, 0.047) | 1  (0.95, 1) | 1.8% | -0.006  (-0.019, 0.0066) | 1  (0.98, 1) | 1.7% | -0.02  (-0.059, 0.019) | 1  (0.94, 1.1) | 2.5% |  |
| N-Acetyl-L-alanine | 0.027  (-0.034, 0.088) | 1  (0.96, 1) | 1.5% | 0.011  (-0.021, 0.044) | 0.98  (0.96, 1) | 1.7% | -0.015  (-0.052, 0.022) | 0.98  (0.96, 1) | 2.3% |  |
| 4-Acetamidobutanoic acid | 0.0017  (-0.001, 0.0043) | 1 (1, 1) | 1.7% | 9.8e-05  (-0.0027, 0.0029) | 0.97  (0.96, 0.98) | 2.4% | -0.0015  (-0.0044, 0.0014) | 0.96  (0.95, 0.97) | 3.4% |  |

## Table S10: Agreement between pairwise sample types, after removing outliers.

Using Deming regression, we estimated intercepts and slopes with their 95% confidence intervals. For each metabolite, we modeled the heparin‐plasma measurement as the dependent variable and the EDTA‐plasma measurement as the independent variable, and we ran analogous models comparing heparin plasma to serum and EDTA plasma to serum. Intercepts close to zero and slopes close to one indicate good agreement. The coefficient of variation (CV %) was calculated by computing each sample’s mean and standard deviation across measurement types to obtain a per‐sample CV (SD/mean), then averaging these per‐sample CVs.Outliers were defined as values more extreme than 3 standard deviations above or below a given marker’s mean value for a given sample type. Here, 1.6% of values across all markers and sample types met the criteria for outliers.

|  | **Heparin Plasma ~ EDTA Plasma** | | | **Heparin Plasma ~ Serum** | | | **EDTA Plasma ~ Serum** | | |
| --- | --- | --- | --- | --- | --- | --- | --- | --- | --- |
|  | **Intercept** | **Slope** | **CV%** | **Intercept** | **Slope** | **CV%** | **Intercept** | **Slope** | **CV%** |
| Pseudouridine | 0.051  (-0.045, 0.15) | 0.99  (0.97, 1) | 1.0% | -0.024  (-0.15, 0.11) | 1  (0.97, 1) | 1.2% | -0.065  (-0.16, 0.03) | 1  (0.98, 1) | 1.0% |
| 2-(alpha-D-Mannopyranosyl)-tryptophan | 0.0055  (-0.006, 0.017) | 0.98  (0.93, 1) | 2.7% | 0.0053  (-0.0077, 0.018) | 0.93  (0.88, 0.98) | 4.7% | 0.0034  (-0.01, 0.017) | 0.93  (0.87, 0.99) | 4.9% |
| SDMA | 0.0031  (-0.025, 0.032) | 1  (0.95, 1.1) | 1.8% | 0.0086  (-0.0086, 0.026) | 0.97  (0.94, 1) | 1.7% | 0.0052  (-0.021, 0.031) | 0.97  (0.92, 1) | 2.2% |
| L-Homocitrulline | 0.0057  (-0.0079, 0.019) | 1  (0.96, 1) | 2.3% | 0.0022  (-0.006, 0.01) | 0.99  (0.97, 1) | 2.1% | -0.0041  (-0.013, 0.0046) | 0.99  (0.97, 1) | 2.4% |
| Kynurenic acid | -0.00043  (-0.0017, 0.00083) | 1  (0.99, 1) | 2.1% | -8.2e-05  (-0.0015, 0.0013) | 0.98  (0.95, 1) | 2.9% | 0.00017  (-0.001, 0.0014) | 0.97  (0.95, 0.99) | 3.1% |
| N2,N2-dimethylguanosine | 0.0012  (-0.00057, 0.0031) | 1  (0.95, 1) | 2.4% | 4e-04  (-0.0018, 0.0026) | 0.98  (0.93, 1) | 2.5% | -0.00091  (-0.0031, 0.0013) | 0.99  (0.94, 1) | 3.0% |
| N-Acetylserine | 0.027  (-0.031, 0.085) | 1  (0.95, 1) | 2.3% | 0.031  (-0.04, 0.1) | 0.97  (0.91, 1) | 2.0% | 0.0029  (-0.035, 0.041) | 0.97  (0.94, 1) | 2.5% |
| N-Acetylneuraminic acid | 0.044  (-0.099, 0.19) | 0.96  (0.77, 1.2) | 7.0% | -0.074  (-0.27, 0.12) | 1.1  (0.82, 1.3) | 5.5% | -0.12  (-0.28, 0.034) | 1.1  (0.93, 1.3) | 8.8% |
| Acetyl-L-threonine | 0.013  (-0.012, 0.038) | 1  (0.97, 1) | 1.8% | -0.0031  (-0.02, 0.014) | 0.99  (0.97, 1) | 1.7% | -0.015  (-0.039, 0.0094) | 0.99  (0.96, 1) | 2.5% |
| N-Acetyl-L-alanine | 0.056  (0.0011, 0.11) | 0.98  (0.95, 1) | 1.5% | 0.024  (-0.024, 0.072) | 0.97  (0.94, 1) | 1.7% | -0.03  (-0.087, 0.026) | 0.99  (0.96, 1) | 2.3% |
| 4-Acetamidobutanoic acid | 0.0011  (-0.0034, 0.0055) | 1  (0.99, 1) | 1.7% | -2e-04  (-0.0045, 0.0041) | 0.98  (0.96, 0.99) | 2.4% | -0.0012  (-0.0063, 0.0039) | 0.96  (0.94, 0.98) | 3.4% |

## Table S11: Comparison of individuals randomly selected versus those not selected from the MESA-Kidney and HIV studies.

|  | **Small error**  **and small**  **eGFRdiff** | | **Small error**  **and large**  **eGFRdiff** | | **Large error**  **and small**  **eGFRdiff** | | **Large error**  **and large**  **eGFRdiff** |
| --- | --- | --- | --- | --- | --- | --- | --- |
| **Overall n** | **277** | | **128** | | **38** | | **51** |
| **Selected for panel eGFR** | **No (n=261)** | **Yes^1^ (n=16)** | **No (n=112)** | **Yes^1^ (n=16)** | **No (n=22)** | **Yes^1^ (n=16)** | **Yes (n=51)** |
| MESA-Kidney participant (%) | 169 (64.8) | 11 (68.8) | 54 (48.2) | 4 (25.0) | 15 (68.2) | 9 (56.2) | 32 (62.7) |
| Age, years (mean (SD)) | 63.22 (13.17) | 64.56 (16.95) | 56.32 (13.56) | 51.31 (12.62) | 61.27 (12.26) | 62.88 (14.25) | 65.37 (16.04) |
| Black race (%) | 142 (54.4) | 7 (43.8) | 48 (42.9) | 10 (62.5) | 8 (36.4) | 11 (68.8) | 17 (33.3) |
| Female sex (%) | 107 (41.0) | 8 (50.0) | 29 (25.9) | 5 (31.2) | 13 (59.1) | 9 (56.2) | 24 (47.1) |
| Diabetes (%) | 46 (17.7) | 1 ( 6.2) | 17 (15.2) | 2 (12.5) | 6 (27.3) | 6 (37.5) | 11 (21.6) |
| BMI, kg/m^2^ (mean (SD)) | 28.43 (5.32) | 28.54 (4.03) | 28.88 (6.51) | 27.00 (6.05) | 27.66 (4.76) | 27.39 (4.80) | 28.97 (7.39) |
| mGFR, ml/min/1.73m^2^ (mean (SD)) | 76.55 (20.74) | 76.75 (25.23) | 86.17 (20.43) | 92.20 (15.28) | 74.35 (29.67) | 82.28 (39.60) | 67.83 (24.35) |
| Serum creatinine, mg/dL (mean (SD)) | 1.05 (0.42) | 1.02 (0.31) | 1.00 (0.24) | 0.99 (0.24) | 1.05 (0.73) | 0.99 (0.46) | 1.05 (0.35) |
| Serum cystatin-c, mg/L (mean (SD)) | 1.05 (0.36) | 0.99 (0.22) | 0.98 (0.27) | 0.97 (0.22) | 1.09 (0.68) | 0.97 (0.24) | 1.20 (0.32) |

^1^Participants were randomly chosen from all those within each given subgroup.

Large error is defined as an absolute percent difference between mGFR and eGFRcr, relative to mGFR, that exceeds 30%; small error is <30%. Large eGFRdiff is defined as an absolute difference in eGFRcr and eGFRcys greater than 15 ml/min/1.73m^2^; small eGFRdiff is < 15 ml/min/1.73m^2^

## Table S12: Comparison of the relationship of the markers with mGFR among untargeted and targeted assays.

Data includes 53 MESA-Kidney samples with measures across all types. Correlations represent the Pearson correlation with mGFR in untargeted data, serum samples, EDTA samples, or Heparin samples.

|  | **Untargeted Assay** | **Serum** | **EDTA** | **Heparin** |
| --- | --- | --- | --- | --- |
| Pseudouridine | -0.60 | -0.76 | -0.75 | -0.74 |
| 2-(alpha-D-Mannopyranosyl)-tryptophan | -0.80 | -0.80 | -0.79 | -0.79 |
| SDMA^1^ | -0.50 | -0.71 | -0.72 | -0.69 |
| L-Homocitrulline | -0.54 | -0.56 | -0.55 | -0.56 |
| Kynurenic acid | -0.40 | -0.44 | -0.45 | -0.44 |
| N2,N2-dimethylguanosine | -0.63 | -0.73 | -0.74 | -0.72 |
| N-Acetylserine | -0.62 | -0.37 | -0.37 | -0.36 |
| N-Acetylneuraminic acid | -0.78 | -0.60 | -0.55 | -0.59 |
| Acetyl-L-threonine | -0.63 | -0.64 | -0.64 | -0.64 |
| N-Acetyl-L-alanine | -0.64 | -0.48 | -0.49 | -0.47 |
| 4-Acetamidobutanoic acid | -0.56 | -0.56 | -0.57 | -0.57 |

^1^Untargeted SDMA includes both SDMA and ADMA, whereas targeted SDMA represents SDMA only

## Figure S1: Creation of clinical validation dataset.

In designing our clinical validation dataset, individuals in the MESA-Kidney and HIV studies were categorized based on whether their eGFRcr error exceeded 30% (versus smaller errors) and/or their eGFRdiff exceeded 15 ml/min/1.73m² (versus smaller differences). Data and specimens were used from all individuals with large errors in eGFRcr and large eGFRdiff Additionally, we randomly sampled 16 random individuals from each of the following groups: those with large errors in eGFRcr and small eGFRdiff, those with small errors in eGFRcr but large eGFRdiff , and those with small errors in eGFRcr but large eGFRdiff.
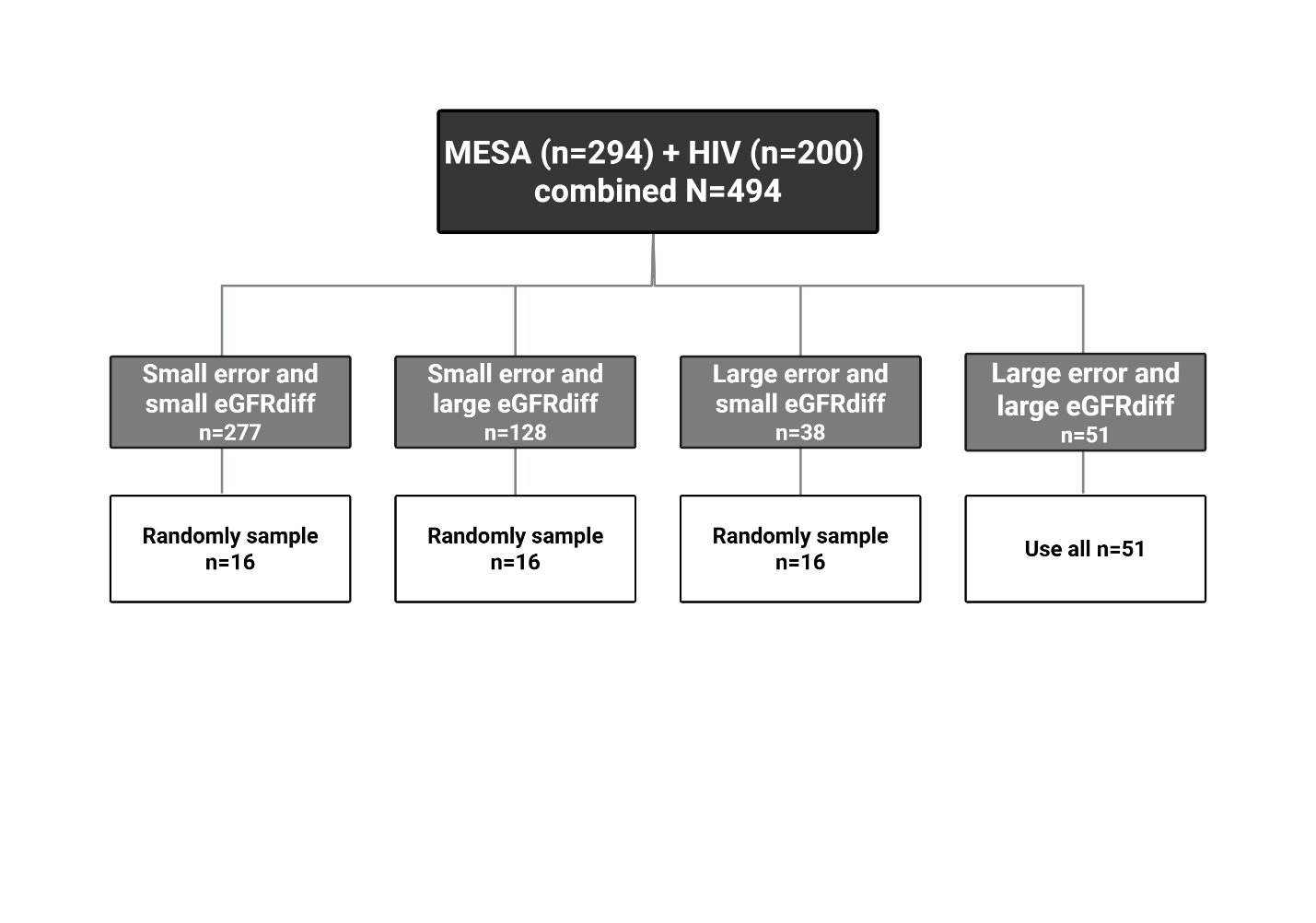


## Figure S2: Representative chromatograms for the lowest calibrator (S1) and a low specimen for each metabolite in a serum specimen.

a: Pseudouridine-S1, b: Pseudouridine-serum, c: 2-(α-D-Mannopyranosyl)-tryptophan-S1, d: 2-(α-D-Mannopyranosyl)-tryptophan-serum, e: SDMA-S1, f: SDMA -serum, g: L-Homocitrulline-S1, h: L-Homocitrulline-serum, i: Kynurenic acid-S1, j: Kynurenic acid-serum, k: N2,N2-dimethylguanosine-S1, l: N2,N2-dimethylguanosine-serum, m: N-Acetylserine-S1, n: N-Acetylserine-serum, o: N-Acetylneuraminic acid-S1, p: N-Acetylneuraminic acid-serum, q: Acetyl-L-threonine-S1, r: Acetyl-L-threonine-serum, s: N-Acetyl-L-alanine-S1, t: N-Acetyl-L-alanine-serum, u: 4-Acetamidobutanoic acid-S1, and v: 4-Acetamidobutanoic acid-serum.

a

g

c

e


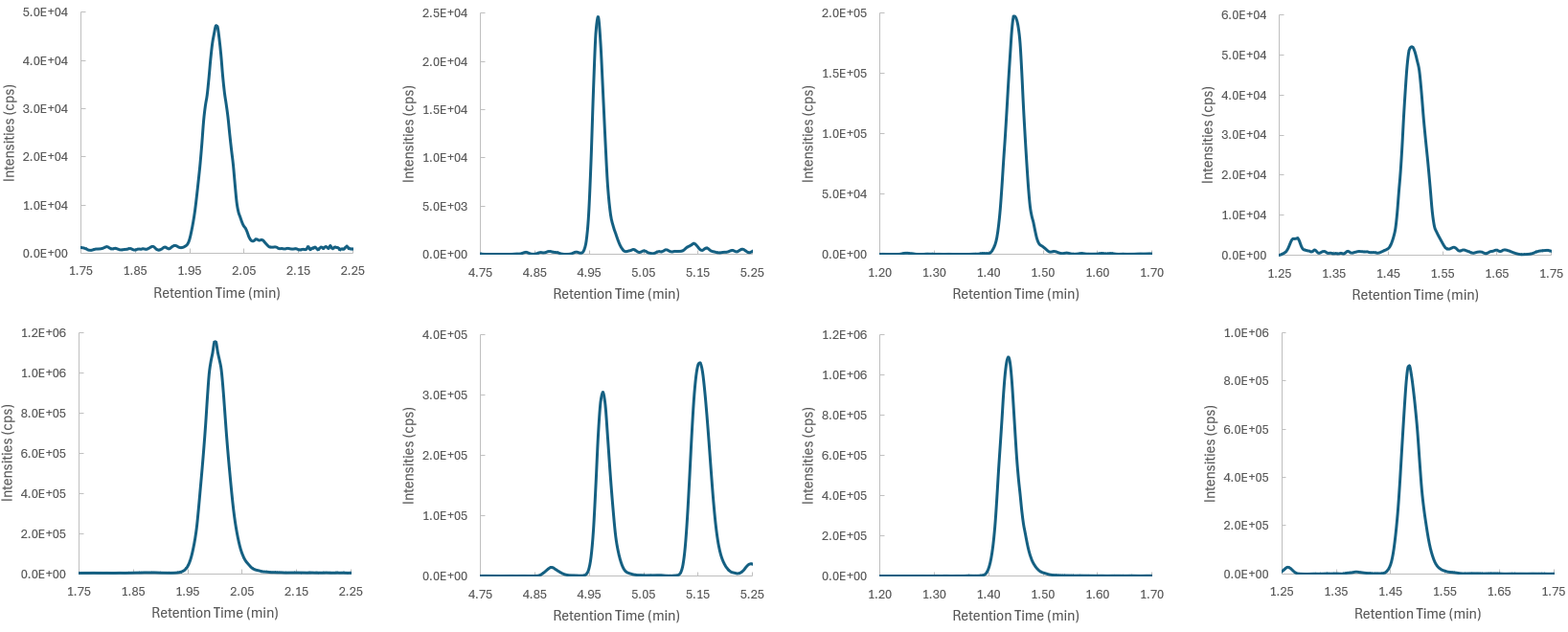


i

o

m

k

h

f

d

b


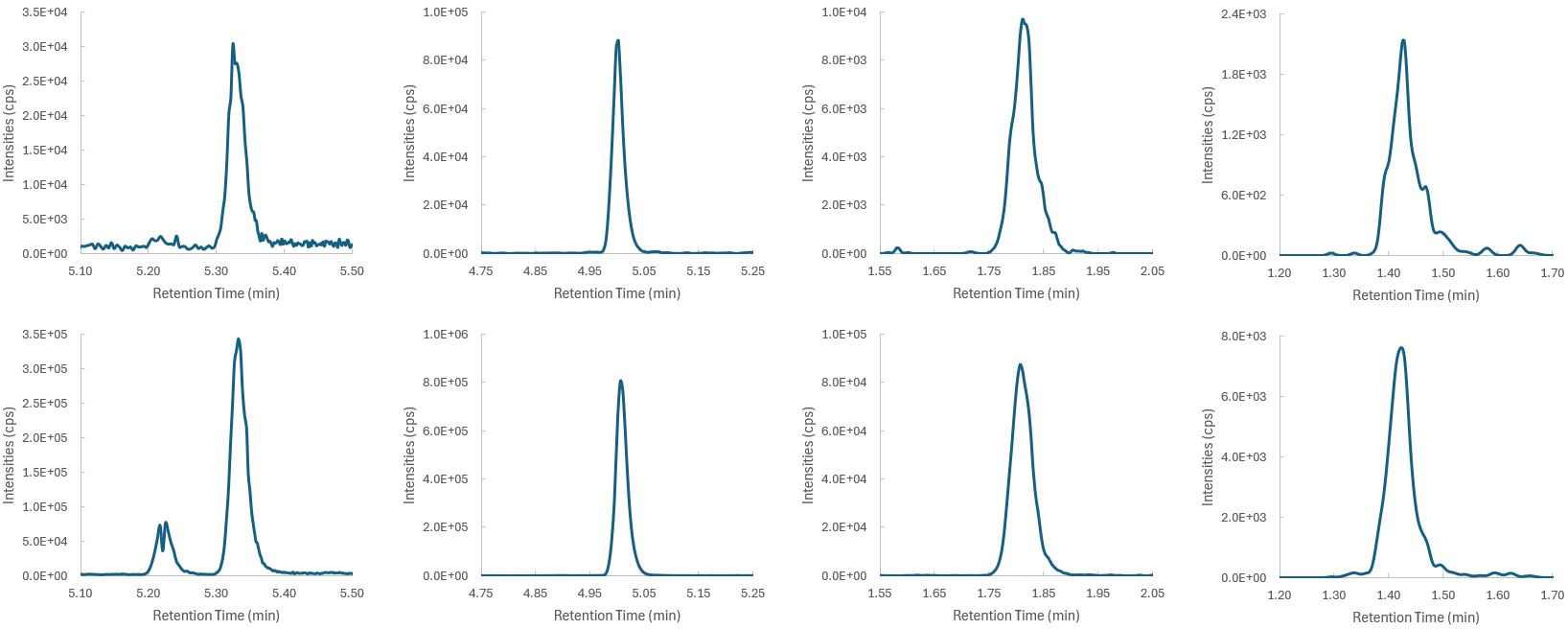


u

s

q

p

n

l

j


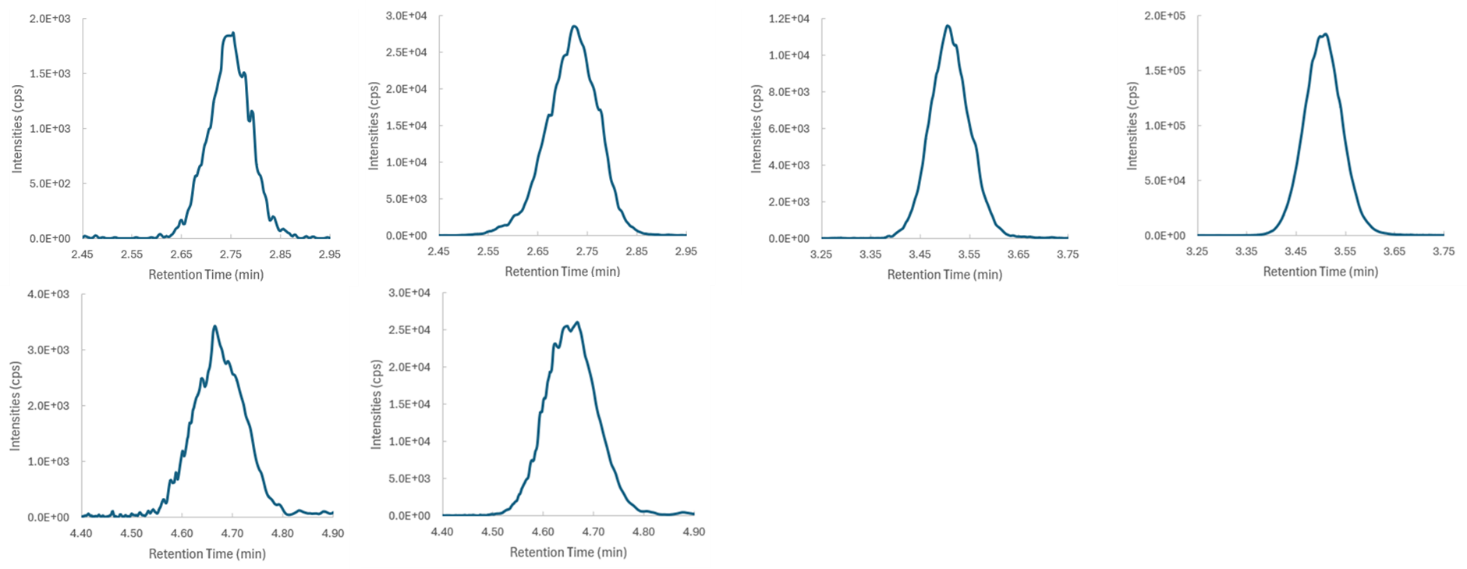

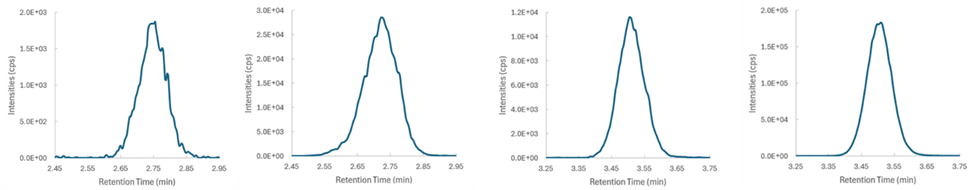

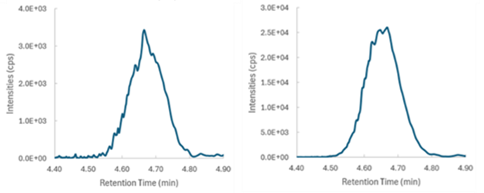


v

t

r


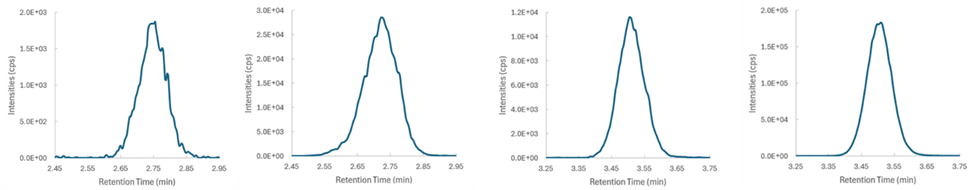

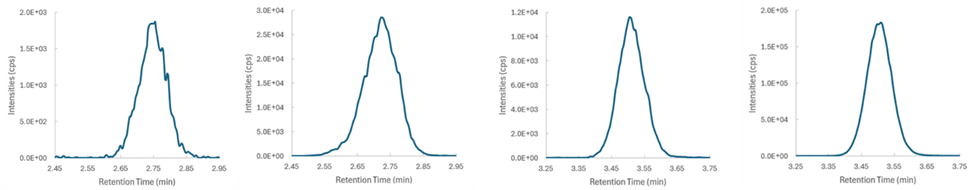

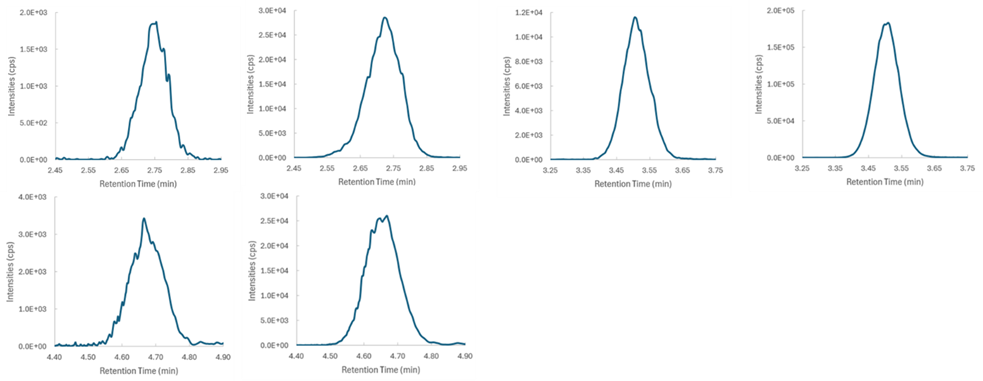


p

## Figure S3: Impact of adding individual demographic terms to single marker panel


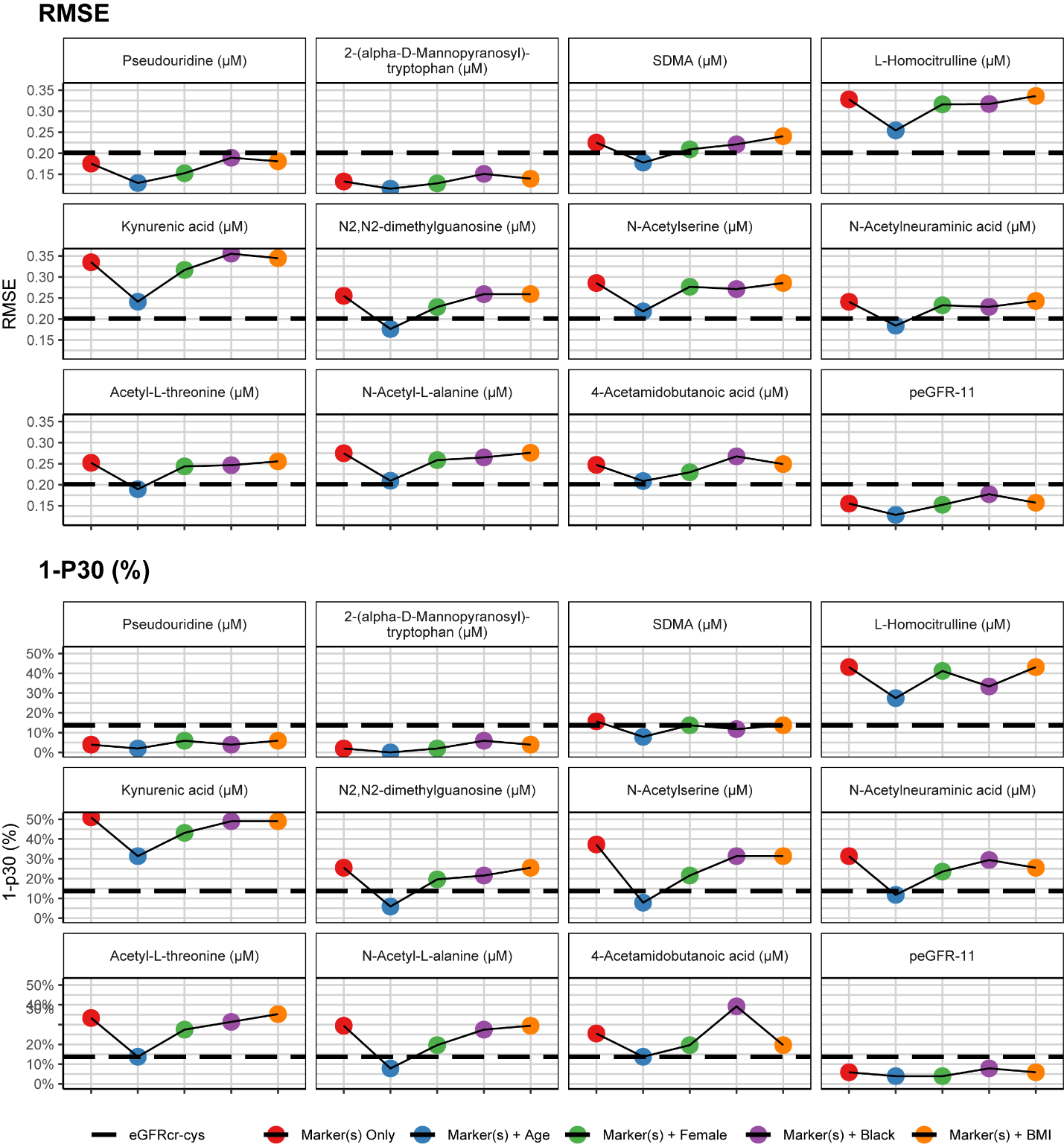


**References**

1. Inker LA, Eckfeldt J, Levey AS, Leiendecker-Foster C, Rynders G, Manzi J, et al. Expressing the CKD-EPI (Chronic Kidney Disease Epidemiology Collaboration) cystatin C equations for estimating GFR with standardized serum cystatin C values. Am J Kidney Dis 2011;58(4):682-4.

2. Inker LA, Wyatt C, Creamer R, Hellinger J, Hotta M, Leppo M, et al. Performance of creatinine and cystatin C GFR estimating equations in an HIV-positive population on antiretrovirals. J Acquir Immune Defic Syndr 2012;61(3):302-9.
